# Gut microbiota and eye diseases: a bibliometric study and visualization analysis

**DOI:** 10.3389/fcimb.2023.1225859

**Published:** 2023-08-09

**Authors:** Xiangyu Fu, Haishan Tan, Ling Huang, Wenyue Chen, Xiang Ren, Danian Chen

**Affiliations:** ^1^ Department of Ophthalmology, West China Hospital, Sichuan University, Chengdu, China; ^2^ Research Laboratory of Ophthalmology and Vision Sciences, State Key Laboratory of Biotherapy, West China Hospital, Sichuan University, Chengdu, China

**Keywords:** gut microbiota, eye disease, inflammation, probiotics, fecal microbial transplantation, bibliometric study, CiteSpace, VOSviewer

## Abstract

**Introduction:**

Recently the role of gut microbial dysbiosis in many ocular disorders, including but not limited to uveitis, age-related macular degeneration (AMD), diabetic retinopathy (DR), dry eye, keratitis and orbitopathy is a hot research topic in the field. Targeting gut microbiota to treat these diseases has become an unstoppable trend. Bibliometric study and visualization analysis have become essential methods for literature analysis in the medical research field. We aim to depict this area's research hotspots and future directions by bibliometric software and methods.

**Methods:**

We search all the related publications from the Web of Science Core Collection. Then, CiteSpace was applied to analyze and visualize the country distributions, dual-map overlay of journals, keyword bursts, and co-cited references. VOSviewer was employed to identify authors, co-cited authors, journals and co-cited journals and display the keyword co-occurrence networks.

**Results:**

A total of 284 relevant publications were identified from 2009 to 2023. The number of studies has been small in the first five years and has grown steadily since 2016. These studies were completed by 1,376 authors from 41 countries worldwide, with the United States in the lead. Lin P has published the most papers while Horai R is the most co-cited author. The top journal and co-cited journal are both Investigative Ophthalmology & Visual Science. In the keyword co-occurrence network, except gut microbiota, inflammation becomes the keyword with the highest frequency. Co-citation analyses reveal that gut dysbiosis is involved in common immune- and inflammation-mediated eye diseases, including uveitis, diabetic retinopathy, age-related macular degeneration, dry eye, and Graves' orbitopathy, and the study of microbiomes is no longer limited to the bacterial populations. Therapeutic strategies that target the gut microbiota, such as probiotics, healthy diet patterns, and fecal microbial transplantation, are effective and critical to future research.

**Conclusions:**

In conclusion, the bibliometric analysis displays the research hotspots and developmental directions of the involvement of gut microbiota in the pathogenesis and treatment of some ocular diseases. It provides an overview of this field's dynamic evolution and structural relationships.

## 1 Introduction

The commensal microbiota is a collective term for microorganisms colonizing the skin or mucous membranes, including the gastrointestinal tract, respiratory tract, oral cavity, conjunctiva, and vagina, most of which are located in the intestine. It is estimated that there are around 10^14^ microorganisms in the gut, the collective genome of which is much larger than the human genome, consisting of bacteria, fungi, viruses, protozoa, and archaea (Sekirov et al., 2010; Szablewski, 2018). Among them, bacterial communities dominate. *Firmicutes* and *Bacteroidetes* are the prominent bacterial phyla; and the rest include *Actinobacteria*, *Proteobacteria*, *Verrucomicrobia*, *Fusobacteria*, and other bacterial phyla (Hu et al., 2016).

As the largest symbiotic microbiota, the intestinal commensals have become indispensable to the human body. They play multiple physiological functions, including promoting food digestion and absorption (Fang et al., 2022), regulating the host’s immune system (Shi et al., 2017), protecting from pathogens (Pickard et al., 2017), synthesizing amino acids, and vitamins (Brunkwall and Orho-Melander, 2017), and metabolizing oral drugs (Kumar et al., 2019). Lots of factors can contribute to changes in the composition of the gut microbiota, including internal factors such as the interaction of gut microbiome with the innate and adaptive immune system, external factors like diet, drug use such as antibiotics, toxin exposure, and various diseases (Gritz and Bhandari, 2015). Under the influence of these factors, intestinal dysbiosis occurs when there is a severe imbalance between beneficial and pathogenic microbes (Robles Alonso and Guarner, 2013). In such a dysbiotic condition, harmful bacteria or conditional pathogenic groups multiply to promote the occurrence of a series of diseases (Diez-Sainz et al., 2021). Currently, dysbiosis of the intestinal microbiota has been reported in various conditions, including inflammatory bowel disease (Gianchecchi and Fierabracci, 2019), ankylosing spondylitis (Ciccia et al., 2017), multiple sclerosis (Berer et al., 2011), Alzheimer’s disease (Hu et al., 2016), and diabetes (Dedrick et al., 2020; Yang et al., 2021).

In recent years, the role of gut microbial dysbiosis in many ocular disorders, including but not limited to uveitis (Kodati and Sen, 2019), age-related macular degeneration (AMD) (Lima-Fontes et al., 2022), diabetic retinopathy (DR) (Thakur et al., 2022), dry eye (Bai et al., 2023b), keratitis (Jayasudha et al., 2018) and orbitopathy (Biscarini et al., 2023), has also attracted more attention from researchers and become a hot research topic. Targeting gut microbiota to assist in treating diseases has become an unstoppable trend. Therapies including antibiotics (Nakamura et al., 2016), probiotics (Kim et al., 2017; Bai et al., 2023a), dietary modifications (Rowan et al., 2017; Beli et al., 2018), and fecal microbial transplantation (FMT) (Watane et al., 2022) have made initial advances in animal models or clinical trials of eye diseases.

Recently, bibliometric study and visualization analysis have become essential methods for literature analysis in the medical research field. Bibliometric analysis can summarize the existing publications and analyze the research structure and quantitative information in a specific research field. Meanwhile, visualization maps can provide the relative contributions from different countries, authors, and journals and the internal correlation between citing and co-cited papers. Consequently, these analyses can outline the current overall framework and show the focus and development trends of the field (Guler et al., 2016; Gu et al., 2021). As stated previously, gut microbiota has been found to be associated with the eye. Therefore, we aim to depict this area’s research hotspots and future directions by bibliometric software and methods.

## 2 Materials and methods

### 2.1 Search strategies and data collection

**Figure 1:**
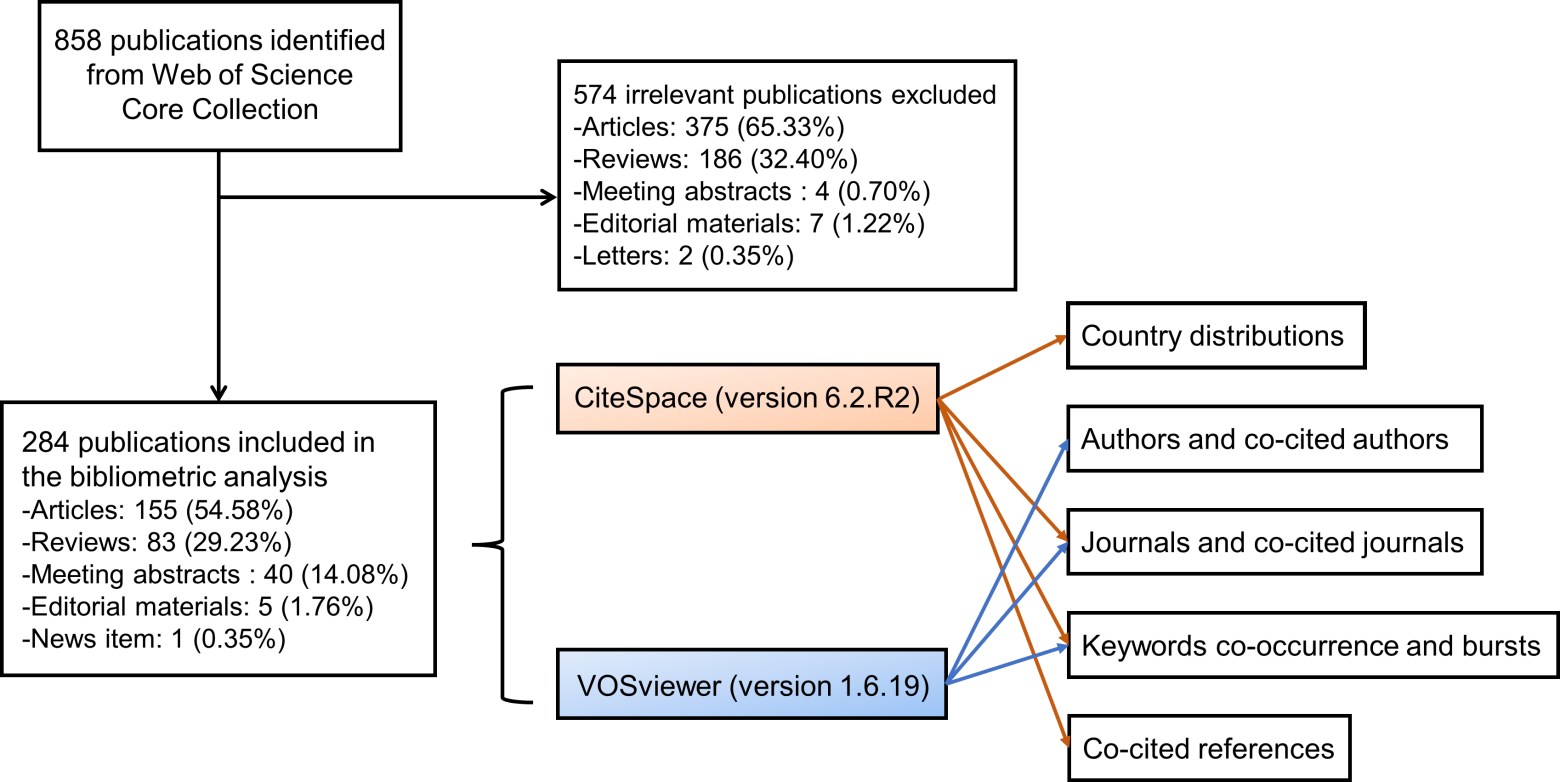
The flow chart of the included publications and methods used in the bibliometric analysis. A total of 284 publications were included in the bibliometric analysis. CiteSpace was applied to analyze and visualize the country distributions, dual-map overlay of journals, keyword bursts, and co-cited references, and VOSviewer was employed to identify authors and co-cited authors, journals and co-cited journals, and to display the keyword co-occurrence networks.

The Web of Science (WoS) Core Collection database was searched for all literature on gut microbiota and ocular diseases. All searches were completed on the same day to avoid bias in the number of documents due to database updates. We broadened the searches by adding some terms of eye diseases that had been reported to be associated with the gut microbiota (Cavuoto et al., 2019). The final retrieval strategies are integrated as follows: TS= (“gut microb*” or “intestinal microb*” or “gut microflora” or “intestinal microflora” or “gut microorganism” or “intestinal microorganism” or “probiotics” or “prebiotics” or “synbiotics”) AND TS= (“eye” or “ocular” or “ophthalm*” or “retin*” or “uveitis” or “keratitis” or “age-related macular degeneration” or “glaucoma” or “orbitopathy”) AND Timespan: 1900-01-01 to 2023-04-03. A total of 858 publications were identified from WoS, and 574 irrelevant publications were excluded after manual screening by reading all titles and abstracts and skimming the full text of some ambiguous documents. Finally, 284 publications were included in the bibliometric analysis, containing 155 articles, 83 reviews, 40 meeting abstracts, 5 editorial materials, and 1 news item (**Figure 1**). Eligible publications were saved and exported as plain text files, including titles, authors, keywords, institutions, countries, publishing journals, references, and citations.

### 2.2 Bibliometric analyses

All exported data were imported into CiteSpace version 6.2.R2 (Drexel University, Philadelphia, United States) (Chen, 2006) and VOSviewer version 1.6.19 (Leiden University, Leiden, Netherlands) (van Eck and Waltman, 2010). We used the “Remove Duplicates” function in CiteSpace to eliminate potentially duplicate records. And then, the synonyms for terms in some areas, such as the countries, keywords, and cited journals, were merged for more accurate results. The citation report of WoS provided the publication and citation trends from 2009 to 2023. CiteSpace was applied to analyze and visualize the country distributions, dual-map overlay of journals, keyword bursts, and co-cited references (**Figure 1**). CiteSpace can perform co-citation analysis on references and obtain cluster view and timeline view through a similarity algorithm so that the history of knowledge evolution or the historical span of documents in a certain cluster will be described in the time dimension, and the development trends of the link between gut microbiota and the eye can be recognized. VOSviewer was employed to identify authors, co-cited authors, journals and co-cited journals and display the keyword co-occurrence networks (**Figure 1**). Co-occurrence analysis can mark keywords in graduated colors based on time course or divide them into clusters with different colors.

## 3 Results

### 3.1 The publication and citation trends

**Figure 2:**
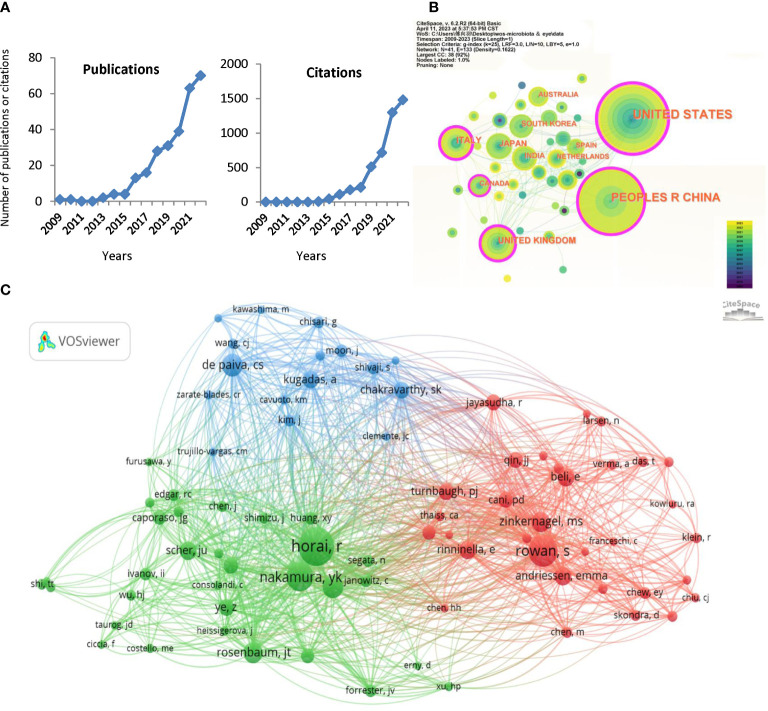
Distribution of publications and citations from different years, countries and authors. **(A)** The citation report of the publication and citation trends from 2009 to 2022. **(B)** Country distributions of the publications. Countries with purple rings on the periphery have a high centrality. **(C)** VOSviewer visualization map of the co-cited authors. VOSviewer automatically classified co-authors with over 15 citations into three sections (the green, red, and blue sections respectively).

The number of publications and citations may reflect the progression and direction of studies in a field, and **Figure 2A** shows the number and trends of publications related to gut microbiota and eye diseases (There were 15 publications and 493 citations in 2023 till April 3, 2023, not shown in **Figure 2A**). It is easy to discover that the number of articles published yearly was fewer than five before 2015, while it has steadily increased since 2016. Especially in the past 2022, the number of publications and citations peaked, with 70 documents and 1480 citations (**Figure 2A**). The 284 publications are cited 4,928 times (3,645 times after removing self-citation) in the WoS database, with an average of 17.4 citations (12.8 citations without self-citation) per publication. This result indicates that the role of gut microbiota in eye diseases has received more and more attention in recent years and is gradually becoming a research focus.

### 3.2 Analysis of leading countries

The published studies are distributed in 41 countries worldwide, and **Table 1** lists the top 5 country distributions of publications. Among them, studies from the United States (97, 34.155%) and the People’s Republic of China (88, 30.986%) each accounted for about one-third of the total, showing a prominent numerical advantage, followed by the United Kingdom (24, 8.451%) and Italy (22, 7.746%) (**Table 1**). In the country distribution network shown in **Figure 2B**, the two core nodes are the United States and the People’s Republic of China. It is worth noting that some nodes with purple rings on the periphery, including the United States, the People’s Republic of China, the United Kingdom, Italy, and Canada (**Figure 2B**), have a high centrality, indicating that researches that made significant contributions to this field or connected several subfields under the topic are mainly from these countries.

**Table 1:** Top 5 country distributions of publications.

Rank	Country	Centrality	Counts (%)	Citations
1	United States	0.55	97 (34.155)	2306
2	Peoples R China	0.40	88 (30.986)	1142
3	United Kingdom	0.32	24 (8.451)	633
4	Italy	0.31	22 (7.746)	545
5	Japan	0.02	17 (5.986)	467

### 3.3 Analysis of authors and co-cited authors

A total of 1,376 authors are involved in the research about the association between gut microbiota and eye disease, and **Table 2** lists the top 10 authors and co-cited authors. Interestingly, more than half of the authors and co-cited authors in the table are American, suggesting that the United States plays a crucial role in the field, consistent with the leading country analysis (**Table 2**). Lin P, Rosenbaum JT, Nakamura YK, and Asquith M are all from Oregon Health and Science University, United States, and often collaborated on papers. At the same time, Horai R and Caspi RR also come from the same laboratory (Laboratory of Immunology, National Eye Institute, National Institutes of Health, USA) and co-authored several related publications. These authors rank highly on the author or co-cited author lists (**Table 2**).

**Table 2:** Top 10 authors and co-cited authors.

Rank	Author	Country	Counts (%)	Co-cited author	Country	Citation counts
1	Lin P	United States	14 (4.930)	Horai R	United States	115
2	Rosenbaum JT	United States	13 (4.577)	Rowan S	United States	88
3	Horai R	United States	12 (4.225)	Nakamura YK	United States	79
4	Asquith M	United States	11 (3.873)	Lin P	United States	58
5	Skondra D	United States	11 (3.873)	de Paiva CS	United States	58
6	Caspi RR	United States	9 (3.169)	Rosenbaum JT	United States	50
7	Shivaji S	India	9 (3.169)	Zinkernagel MS	Switzerland	49
8	Grant MB	United States	8 (2.817)	Andriessen EMMA	Canada	44
9	Huang XY	Peoples R China	7 (2.465)	Rinninella E	Italy	44
10	Kim MK	South Korea	7 (2.465)	Scher JU	United States	43

VOSviewer automatically classified co-authors with over 15 citations into three sections (**Figure 2C**). The green section, centered on Horai R and Nakamura YK, focuses on ocular autoimmunity and autoimmune uveitis (Nakamura et al., 2017; Horai and Caspi, 2019). Rowan S and Zinkernagel MS, who are represented by well-marked red nodes, aim to explore the association between gut microbiota, diet, and AMD (Zinkernagel et al., 2017; Rowan et al., 2021). de Paiva CS and Kugadas A occupy a prominent position in the blue part and are known for their research directions, such as Sjogren’s syndrome, ocular surface mucosal barrier, and ocular surface inflammation (de Paiva et al., 2016; Kugadas et al., 2017). The enrichment and link of co-authors suggest the specific research basis and progress of gut microbiota in ophthalmology.

### 3.4 Analysis of journals and co-cited journals

**Figure 3:**
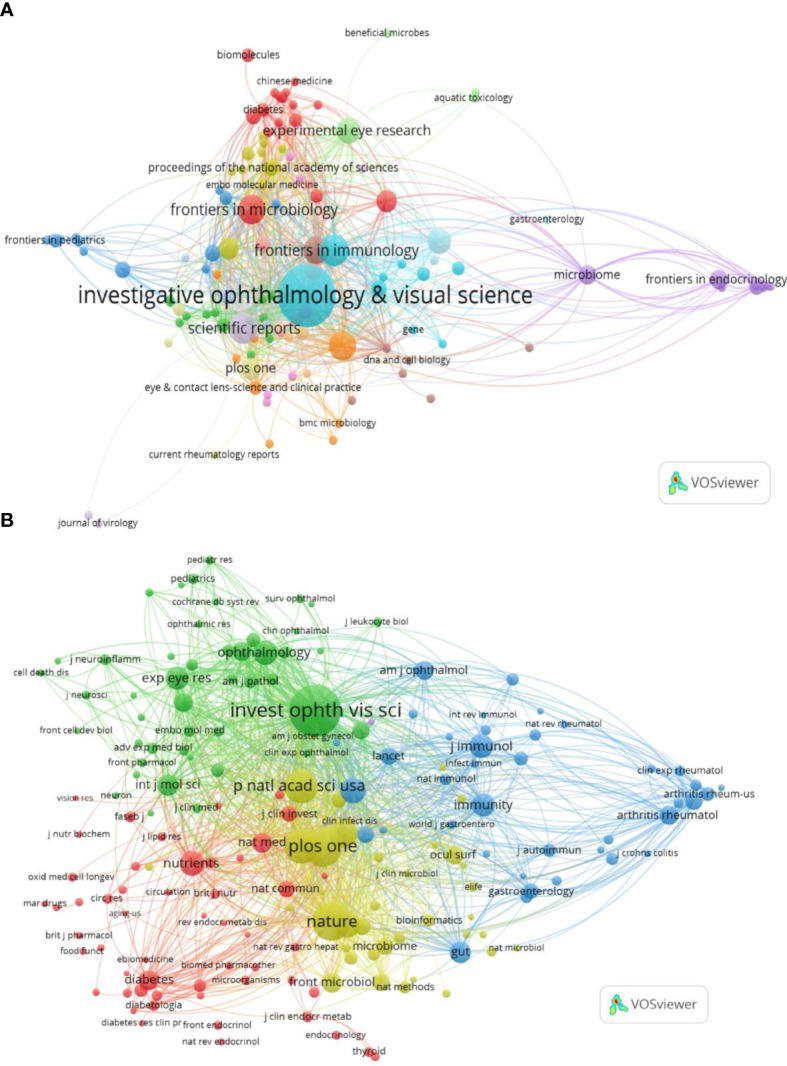
Distribution of publications and citations from different journals. Visualization maps of the journals **(A)** and co-cited journals **(B)**. Journals with the more publications or the higher co-citation frequency are symbolized as the larger nodes. The top journal and co-cited journal are both *Investigative Ophthalmology & Visual Science*.

The collected papers are published in 136 journals, of which *Investigative Ophthalmology & Visual Science (IOVS)* is the leading journal published the most papers (40, 14.085%), followed by *Scientific Reports* (10, 3.521%), *Frontiers in Immunology* (10, 3.521%), *International Journal of Molecular Sciences* (9, 3.169%), and *Frontiers in Microbiology* (9, 3.169%) (**Table 3**; **Figure 3A**). These are well-known journals in ophthalmology, immunology, microbiology, and multidisciplinary science.

**Table 3:** Top 11 journals and co-cited journals.

Rank	Journal	Counts (%)	JCR (2022)	Co-cited journal	Citation counts	JCR (2022)
1	*Investigative Ophthalmology & Visual Science*	40 (14.085)	Q1	*Investigative Ophthalmology & Visual Science*	786	Q1
2	*Scientific Reports*	10 (3.521)	Q2	*PLoS One*	483	Q2
3	*Frontiers in Immunology*	10 (3.521)	Q1	*Nature*	482	Q1
4	*International Journal of Molecular Sciences*	9 (3.169)	Q1	*Scientific Reports*	386	Q2
5	*Frontiers in Microbiology*	9 (3.169)	Q2	*Proceedings of the National Academy of Sciences of the United States of America*	367	Q1
6	*Nutrients*	8 (2.817)	Q1	*Science*	282	Q1
7	*Experimental Eye Research*	7 (2.465)	Q2	*Frontiers in Immunology*	236	Q1
8	*Frontiers in Cellular and Infection Microbiology*	7 (2.465)	Q1	*Cell*	216	Q1
9	*PLoS One*	5 (1.761)	Q2	*Ophthalmology*	215	Q1
10	*Frontiers in Cell and Developmental Biology*	5 (1.761)	Q1	*Nutrients*	213	Q1
11	*Journal of Clinical Medicine*	5 (1.761)	Q2	*Journal of Immunology*	203	Q2

Q1: Quartile 1 of JCR 2022.

Co-citation analysis of journals can reveal the strength of associations between journals or articles. In general, the higher the co-citation frequency of a journal is, the greater its influence in the field. **Table 3** also displays the top co-cited journals that have been cited more than 200 times. In line with published journals, *IOVS* (786 times) remains at the top of the list, accompanied by comprehensive journals such as *Plos One* (483 times) and *Nature* (482 times). Similarly, in the visualization analysis, VOSviewer clearly divided co-cited journals with more than 20 citations into four clusters (**Figure 3B**). The green cluster, where *IOVS* is regarded as the largest node, represents ophthalmology journals, including important journals like *Ophthalmology* and *Experimental Eye Research*. The red section is mostly made up of journals closely related to nutrition and metabolism, such as *Nutrients* and *Diabetes*. The yellow zone has several nodes and includes highly cited comprehensive journals such as *Nature*, *Proceedings of the National Academy of Sciences of the United States of America (PNAS)*. The blue part is mainly correlated with the field of immunology, with *Immunity* as the representing journal.

The journal impact factor (IF) is also one of the indicators of a journal’s impact and significance in particular fields, calculated as the average citation counts of the journal’s publications in a specific year. According to IF 2022, IF of *Frontiers in Immunology* (7.3) is prominent among the top 11 published journals, and *Nature* has the highest IF (64.8) in the top 11 co-cited journals. Moreover, in terms of the journal citation reports (JCR) in 2022 (Clarivate, United Kingdom), most of the leading journals and co-cited journals are listed in Quartile 1 (Q1), and no journals are in Q3 or Q4 (**Table 3**).

**Figure 4:**
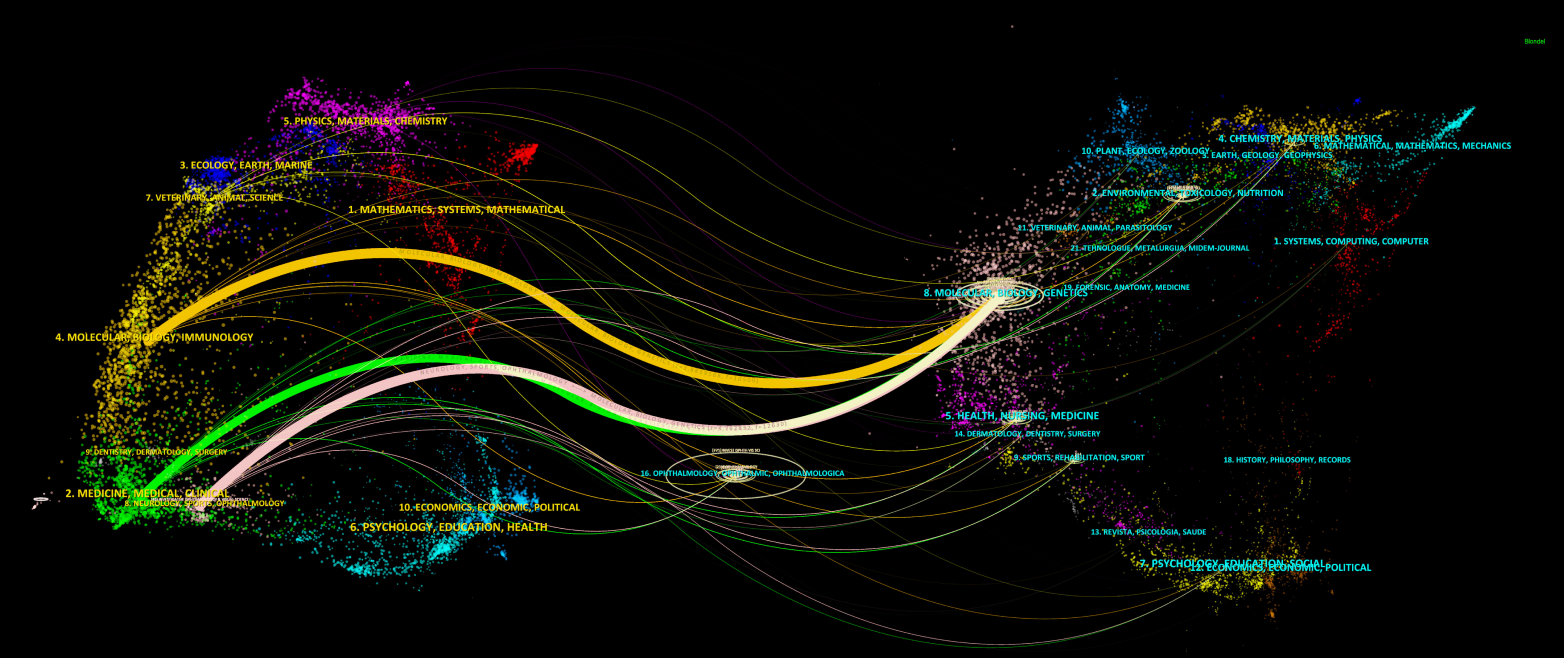
The dual-map overlay of journals. The dual-map overlay of journals displays the associations between publications and citations, with dots representing citing journals in the left and cited journals in the right, so that the citation relationships are depicted as colored lines from the left to the right.

Simultaneously, CiteSpace was used to connect the citing journals and cited journals and show their correspondence in the dual-map overlay of journals (Chen et al., 2014). The left side represents citing journals, and the right side indicates cited journals, so the citation relationships are depicted as colored lines from the left to the right. There are three main citation paths, containing one orange path, one green path, and one pink path, respectively (**Figure 4**). Notably, all three tracks end in Molecular/Biology/Genetics journals. That means, studies published in Molecular/Biology/Immunology journals, Medicine/Medical/Clinical journals, and Neurology/Sports/Ophthalmology journals, generally cited papers in Molecular/Biology/Genetics journals (**Figure 4**).

### 3.5 Analysis of co-occurring keywords and burst terms

To a certain extent, the analysis of keywords can demonstrate the hotspots and focus of this research field. Before analyzing, we merged some terms with the same meaning, including synonyms (e.g., “gut microbiota” and “intestinal microbiota”), different expressions (e.g., “microbiota” and “microbiome”), and singular and plural forms (“risk-factor” and “risk-factors”). The top 25 keywords are presented in **Table 4**, which can be further divided into three main categories. The first category is related to “gut” or “microbiota”, such as gut dysbiosis and probiotics. The second category concerns ocular diseases, such as autoimmune uveitis and diabetic retinopathy, and related ophthalmic terms like the retina. The last involves pathogenic processes and mechanisms, including inflammation (e.g., inflammation, oxidative stress), immunity (e.g., T cells, autoimmunity), and metabolism (e.g., obesity), implying that the above processes or related pathways may also be important targets for intervening with the gut microbiota in the treatment of ocular diseases.

**Table 4:** Top 25 keywords.

Rank	Keywords	Counts
1	Gut microbiota	193
2	Inflammation	62
3	Disease	39
4	Gut dysbiosis	36
5	Autoimmune uveitis	36
6	Diabetic retinopathy	34
7	Probiotics	33
8	Age-related macular degeneration	29
9	Obesity	28
10	Mouse model	28
11	T cells	26
12	Activation	24
13	Association	22
14	Autoimmunity	21
15	Dry eye	21
16	Retina	20
17	Cells	20
18	Macular degeneration	18
19	Ocular diseases	18
20	Pathogenesis	17
21	Bacteria	17
22	Oxidative stress	17
23	Gut-retina axis	16
24	Health	16
25	Risk-factors	16

**Figure 5:**
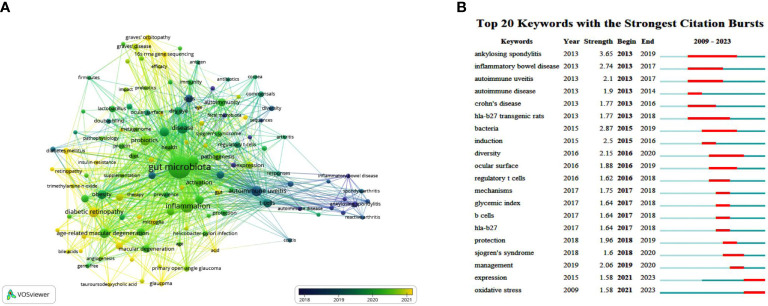
The main keywords. **(A)** Keyword co-occurrence networks. The node size indicates the frequency of keyword occurrence, and the lines connecting nodes represent the strength of the link between keywords. Color mapping by the average year of keyword occurrence is employed to analyze the evolution of research trends. **(B)** The top keywords with the strongest citation bursts. The long blue line depicts the timeline (2009-2023), and the short red line indicates the burst period of certain keyword.

**Figure 5A** shows the keyword co-occurrence networks, where color mapping by the average year of keyword occurrence is employed to analyze the evolution of research trends. This network diagram shows that before the average year of 2018, gut microbiota was originally used to study autoimmune diseases such as ankylosing spondylitis and inflammatory bowel disease. The focus then slowly shifted to autoimmune uveitis, which is marked by a prominent node in the network. This node may lie in the fact that ankylosing spondylitis is frequently comorbid with immune-mediated uveitis, which is also considered the primary ocular manifestation of systemic immune diseases, such as Behcet’s disease and Vogt-Koyanagi-Harada disease (Fu et al., 2021), making the “gut-eye” association begin to attract the attention of researchers. Subsequently, Sjogren’s syndrome and dry eye disease, also mediated by autoimmunity, were gradually appreciated in this topic. As research advanced, the role of gut microbiota in other inflammatory and immune-related eye diseases, including DR, AMD, and Graves’ orbitopathy (GO), has been constantly reported in recent years (especially after 2021) (**Figure 5A**).

The top 20 keywords with the strongest citation bursts generated by CiteSpace are illustrated in **Figure 5B**. Like the keyword co-occurrence network, the burst keywords can reflect the evolution process and development trend of the studies, providing reference and experience for future research. These burst terms can be broadly divided into several categories, including immune-mediated extraocular/systemic diseases (ankylosing spondylitis, inflammatory bowel disease, autoimmune disease, Crohn’s disease, and Sjogren’s syndrome), ocular-related (autoimmune uveitis, ocular surface), immune/inflammatory-related (HLA-B27 transgenic rats, induction, regulatory T cells, B cell, HLA-B27, and oxidative stress), microbial-related (bacteria, diversity), and treatment-related (protection, management). In the early years (2013-2017), multiple autoimmune diseases and intestinal inflammatory diseases suddenly emerged. Since 2015, “bacteria” and “diversity”, which mean the composition and properties of gut microbiota, have been mentioned. Following that, “regulatory T cells”, “HLA-B27”, “ocular surface”, and “Sjogren’s syndrome” became bursts (**Figure 5B**). This trend is consistent with the keyword co-occurrence analysis, indirectly revealing the essential part of gut microbiota in the pathogenesis and therapeutics of immune and inflammatory diseases.

### 3.6 Analysis of co-cited references

#### 3.6.1 Top co-cited references

Cited references are the theoretical basis and knowledge framework of a scientific research subject. If two references are simultaneously cited by one paper, their contents may be related. The more times they are co-cited, the stronger the correlation is. Therefore, statistical analysis of co-cited references is instructive. We count the top 10 co-cited references in **Table 5**. The first two are both AMD-related studies. The most commonly cited reference reported a lower-glycemia diet altered gut microbiota and microbial co-metabolites, thus protecting against the features of AMD in a wild-type aged-mouse model (Rowan et al., 2017). Metagenomic sequencing found alterations in gut microbiome between AMD patients and controls (Zinkernagel et al., 2017). Also worth noting, the third co-cited reference, which contributed to the treatment of DR, confirmed that intermittent fasting could reconstruct intestinal flora composition and improve bile acid metabolism to prevent retinopathy in db/db mice (Beli et al., 2018).

**Table 5:** Top 10 co-cited references.

Rank	Citation counts	Author	Reference title	Journal	Year
1	52	Rowan S	Involvement of a gut-retina axis in protection against dietary glycemia-induced age-related macular degeneration	*P Natl Acad Sci USA*	2017
2	44	Zinkernagel MS	Association of the intestinal microbiome with the development of neovascular age-related macular degeneration	*Sci Rep*	2017
3	42	Beli E	Restructuring of the gut microbiome by intermittent fasting prevents retinopathy and prolongs survival in db/db mice	*Diabetes*	2018
4	41	Nakamura YK	Gut microbial alterations associated with protection from autoimmune uveitis	*Invest Ophth Vis Sci*	2016
5	39	Horai R	Microbiota-dependent activation of an autoreactive T cell receptor provokes autoimmunity in an immunologically privileged site	*Immunity*	2015
6	29	Rinninella E	The role of diet, micronutrients and the gut microbiota in age-related macular degeneration: new perspectives from the gut-retina axis	*Nutrients*	2018
7	29	Nakamura YK	Short chain fatty acids ameliorate immune-mediated uveitis partially by altering migration of lymphocytes from the intestine	*Sci Rep*	2017
8	29	de Paiva CS	Altered mucosal microbiome diversity and disease severity in sjogren syndrome	*Sci Rep*	2016
9	27	Andriessen EMMA	Gut microbiota influences pathological angiogenesis in obesity-driven choroidal neovascularization	*Embo Mol Med*	2016
10	27	Huang XY	Gut microbiota composition and fecal metabolic phenotype in patients with acute anterior uveitis	*Invest Ophth Vis Sci*	2018

#### 3.6.2 Eight clusters of the co-citation network

**Figure 6:**
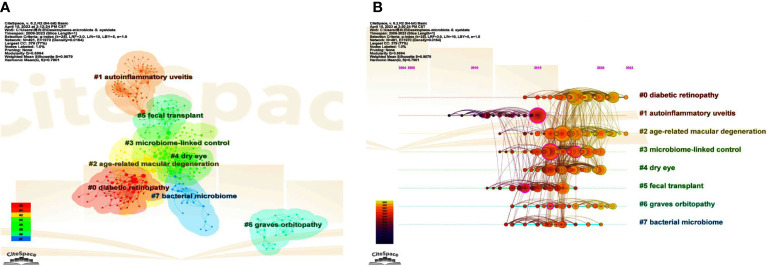
The main co-citation clusters. **(A)** CiteSpace visualization clusters of the co-cited references. Terms from the title field of the citing papers within each cluster are used as the definition of that cluster. **(B)** Timeline view of these listed clusters of the co-cited references.

The co-citation network can be carved into different clusters according to the log-likelihood ratio (LLR) algorithm using CiteSpace, and the cited papers from the same cluster are much more closely related. Terms from the title field of the citing documents within each cluster are used to define that cluster. We can find the top 8 clusters in **Figure 6A**, which are #0 diabetic retinopathy, #1 autoinflammatory uveitis, #2 age-related macular degeneration, #3 microbiome-linked control, #4 dry eye, #5 fecal transplant, #6 graves’ orbitopathy, and #7 bacterial microbiome, respectively. Five of these clusters (#0, #1, #2, #4, and #6) are about various eye diseases, whereas the other three (#3, #5, and #7) focus on aspects of gut microbiota. Among them, co-cited references in cluster #3 describe the control of immune homeostasis by the gut microbiome and the regulation of the microbiome on autoimmune states, primarily on uveitis, suggesting a close relationship with cluster #1.

#### 3.6.3 Timeline map of clusters

A cluster map can be converted to a timeline view to observe research dynamics and progressions in the listed clusters over the timeline. Of note, autoinflammatory uveitis, also known as autoimmune uveitis, was first used to study associations with gut microbiota (**Figure 6B**). Consistent with what was mentioned above, cluster #3, which has many apparent links with cluster #1, can be regarded as a continuation of #1 over time. Interestingly, studies related to FMT (cluster #5), a meaningful way to verify the causality of the intestinal microbiome or the effectiveness of therapies by modifying the microbiota, stagnated around 2018, indicating that the use of FMT in ocular diseases is still minimal (**Figure 6B**). In contrast, microbiome-linked studies about DR and AMD have continued until recently (**Figure 6B**).

#### 3.6.4 High betweenness centrality papers

In the timeline map of clusters (**Figure 6B**), several nodes are marked by purple rings, representing a high “betweenness centrality”. With higher betweenness centrality, these references act as vital bridges connecting different subfields. **Table 6** displays the top 8 references with the highest “betweenness centrality” among the top 8 clusters, most of which are from clusters #1 and #3, highlighting the importance of some immune processes, such as molecular mimicry and immune cells, such as regulatory T cells, in the involvement of gut microbiota in autoimmune diseases. The remaining two papers are from clusters #6 and #5. In particular, the article from cluster #6 (graves’ orbitopathy) (Berchner-Pfannschmidt et al., 2016) has the highest betweenness centrality in all co-cited papers, which assessed a TSHR A-subunit plasmid-immunized preclinical model of GO in female BALB/c mice under different environments, and may provide great convenience and strong support for the study on the association between GO with the intestine microbiome.

**Table 6:** Cited references with the highest “betweenness centrality” among the top 8 clusters.

Rank	Centrality	References	Cluster #
1	0.17	Berchner-Pfannschmidt U (2016) Comparative assessment of female mouse model of graves' orbitopathy under different environments, accompanied by proinflammatory cytokine and t-cell responses to thyrotropin hormone receptor antigen	6
2	0.16	Avni O (2018) Molecular (Me)micry?	3
3	0.15	Nakamura YK (2016) Gut microbial alterations associated with protection from autoimmune uveitis	3
4	0.14	Huang XY (2018) Gut microbiota composition and fecal metabolic phenotype in patients with acute anterior uveitis	3
5	0.14	Lin P (2014) HLA-B27 and human beta 2-microglobulin affect the gut microbiota of transgenic rats	5
6	0.13	Horai R (2015) Microbiota-dependent activation of an autoreactive T cell receptor provokes autoimmunity in an immunologically privileged site	1
7	0.12	Atarashi K (2013) T-reg induction by a rationally selected mixture of *Clostridia* strains from the human microbiota	1
8	0.11	Arnold IC (2011) *Helicobacter pylori* infection prevents allergic asthma in mouse models through the induction of regulatory T cells	1

#### 3.6.5 Details of cluster #1 (autoinflammatory uveitis) and #3 (microbiome-linked control)

Clusters #1 and #3 are related to microbiome-linked control over autoimmune and autoinflammatory uveitis (**Table 7**). Except for one clinical research from China (Huang et al., 2018) and a review article (Horai and Caspi, 2019), the remaining top-cited references of the two clusters are all animal experiments. Nakamura YK, the third top co-cited author in all references (**Table 2**), is one of the authors of two animal studies that established B10RIII mouse model of induced experimental autoimmune uveitis (EAU) by active immunization with inter-photoreceptor retinoid-binding protein (IRBP) emulsified in the complete Freund’s adjuvant (Nakamura et al., 2016; Janowitz et al., 2019). In contrast, another prominent cited author, Horai R, who ranks top in the co-cited author list (**Table 2**), used a novel model of spontaneous uveitis. The spontaneously uveitic R161H mouse could express an IRBP-specific T cell receptor transgene on the B10.RIII background (Horai et al., 2015). As for the citing articles, all these seven publications belong to review articles, two of which were written by Rosenbaum JT (**Table 7**) (Rosenbaum and Kim, 2013; Rosenbaum et al., 2016), an author who has made a significant contribution to this field, as previously stated.

**Table 7:** Cited references and citing articles of cluster #1 autoinflammatory uveitis and #3 microbiome-linked control.

Clusters	Cited references		Citing articles	
	Author (year) journal, volume	Citation counts	Author (year) title	Coverage counts
#1 Autoinflammatory uveitis				
	Horai R (2015) Immunity, 43	39	Rosenbaum JT (2013) Innate immune signals in autoimmune and autoinflammatory uveitis	26
	Atarashi K (2011) Science, 331	6	Consolandi C (2015) Behcet’s syndrome patients exhibit specific microbiome signature	19
	Berer K (2011) Nature, 479	6	Rosenbaum JT (2016) The microbiome, HLA, and the pathogenesis of uveitis	10
#3 Microbiome-linked control				
	Nakamura YK (2016) Invest Ophth Vis Sci, 57	41	Moon J (2020) Can gut microbiota affect dry eye syndrome?	34
	Huang XY (2018) Invest Ophth Vis Sci, 59	27	Xue W (2021) Microbiota and ocular diseases	24
	Janowitz C (2019) Invest Ophth Vis Sci, 60	25	Fu X (2021) The role of gut microbiome in autoimmune uveitis	20
	Horai R (2019) Front Immunol, 10	23	Baim AD (2019) The microbiome and ophthalmic disease	17

#### 3.6.6 Details of cluster #0 (diabetic retinopathy), #2 (age-related macular degeneration), #4 (dry eye), and #6 (graves’ orbitopathy)

In addition to uveitis, common eye diseases, including DR, AMD, dry eye, and GO, have been reported to be closely associated with intestinal microbiota (Moon et al., 2020c; Jiao et al., 2021; Zhang et al., 2022; Li et al., 2023). In cluster #0 (diabetic retinopathy) (**Table 8**), the top-cited study conducted on db/db mice and published on *Diabetes*, explored how intermittent fasting altered the composition of gut microbiota and consequently reduced DR severity (Beli et al., 2018). Its influence goes far beyond other references within the cluster, laying the groundwork for studying the role of microbiota in DR. Moreover, the clinical research conducted by Das T et al. (Das et al., 2021), the citing article, and the cited reference, compared alterations in gut bacterial microbiome among DR, diabetes mellitus, and healthy control groups.

**Table 8:** Cited references and citing articles of cluster #0 diabetic retinopathy, #2 age-related macular degeneration, #4 dry eye, and #6 graves’ orbitopathy.

Clusters	Cited references		Citing articles	
	Author (year) journal, volume	Citation counts	Author (year) title	Coverage counts
#0 Diabetic retinopathy				
	Beli E (2018) Diabetes, 67	42	Nadeem U (2022) Gut microbiome and retinal diseases: an updated review	23
	Chakravarthy SK (2018) Indian J Microbiol, 58	26	Bringer M (2021) The gut microbiota in retinal diseases	21
	Das T (2021) Sci Rep, 11	22	Das T (2021) Alterations in the gut bacterial microbiome in people with type 2 diabetes mellitus and diabetic retinopathy	15
	Huang YH (2021) Front Cell Infect Mi, 11	20	Jiao J (2021) Recent insights into the role of gut microbiota in diabetic retinopathy	14
#2 Age-related macular degeneration				
	Rowan S (2017) P Natl Acad Sci USA, 114	52	Moon J (2020) Can gut microbiota affect dry eye syndrome?	38
	Zinkernagel MS (2017) Sci Rep, 7	44	Xue W (2021) Microbiota and ocular diseases	32
	Rinninella E (2018) Nutrients, 10	29	Scuderi G (2022) Gut microbiome in retina health: the crucial role of the gut-retina axis	18
	Andriessen EMMA (2016) Embo Mol Med, 8	27	Pezzino S (2023) Microbiome dysbiosis: a pathological mechanism at the intersection of obesity and glaucoma	15
#4 Dry eye				
	de Paiva CS (2016) Sci Rep, 6	29	Moon J (2020) Can gut microbiota affect dry eye syndrome?	27
	Moon J (2020) *PLoS One*, 15	19	Moon J (2020) Effect of IRT5 probiotics on dry eye in the experimental dry eye mouse model	15
	Kugadas A (2017) Invest Ophth Vis Sci, 58	17	Moon J (2020) Gut dysbiosis is prevailing in Sjogren’s syndrome and is related to dry eye severity	14
	Lin P (2018) Curr Opin Ophthalmol, 29	17	Baim AD (2019) The microbiome and ophthalmic disease	12
	Kim J (2017) Nutrients, 9	15	Schaefer L (2022) Gut microbiota from Sjogren syndrome patients causes decreased T regulatory cells in the lymphoid organs and desiccation-induced corneal barrier disruption in mice	12
#6 Graves’ orbitopathy				
	Su XH (2020) J Clin Endocr Metab, 105	8	Virili C (2021) Gut microbiome and thyroid autoimmunity	10
	Masetti G (2018) Microbiome, 6	7	Hou J (2021) The role of the microbiota in Graves’ disease and Graves’ orbitopathy	7
	Ishaq HM (2018) Int J Biol Sci, 14	7	Li Y (2022) The role and molecular mechanism of gut microbiota in Graves’ orbitopathy	7

Papers from Rowan S et al. (Rowan et al., 2017) and Zinkernagel MS et al. (Zinkernagel et al., 2017) are the most cited references in cluster #2 (age-related macular degeneration) (**Table 8**), also in all clusters (**Table 5**), indicating the potential role of gut microbes in AMD has attracted public attention. Diet and obesity have been identified as vital environmental risk factors for AMD, and diet is one of the critical factors in changing the gut microbiota (Nadeem et al., 2022). Therefore, the impact of dietary patterns such as high-fat diet (Andriessen et al., 2016) and a high-glycemia diet (Rowan et al., 2017) on AMD has been extensively studied, and micronutrient intake has also been reviewed (Rinninella et al., 2018; Cao et al., 2022). Interestingly, clusters #2 and #4 (dry eye) share the same citing article with the most coverage (Moon et al., 2020c). The majority of the articles from cluster #4 are about the regulation of ocular surface health and ocular surface diseases such as Sjogren’s syndrome and dry eye syndrome (de Paiva et al., 2016; Kugadas et al., 2017; Schaefer et al., 2022b). And over half of these representative citing articles in this section (**Table 8**) were completed by Moon J and colleagues, including two original articles and one review (Moon et al., 2020a; Moon et al., 2020b; Moon et al., 2020c). Of note, IRT5, a mixed probiotic consisting of *Lactobacillus casei*, *Lactobacillus acidophilus*, *Lactobacillus reuteri*, *Bifidobacterium bifidum*, and *Streptococcus thermophilus*, was mentioned to potentially decrease the severity of experimental dry eye model, which indicated that probiotics might be an effective means to treat disease by intervening with the gut microbiota (Kim et al., 2017; Moon et al., 2020b). In contrast, there is less attention to cluster #6 (graves’ orbitopathy), which is isolated from other clusters in the cluster map (**Figure 6A**). The cited references are primarily about Graves’ disease (GD) (Ishaq et al., 2018; Su et al., 2020), followed by GO (Masetti et al., 2018), while the citing articles are reviews relevant to the field (Hou et al., 2021; Virili et al., 2021; Li et al., 2023) (**Table 8**).

#### 3.6.7 Details of cluster #5 (fecal transplant) and #7 (bacterial microbiome)

Clusters #5 (fecal transplant) and #7 (bacterial microbiome) focus on characterizing the gut microbiome (**Table 9**). In the citing reviews, FMT is deemed as a potentially effective therapeutic strategy to spondyloarthritis and uveitis by replacing the gut microbiome with a normal one (Choi et al., 2018; Rosenbaum and Asquith, 2018), although it has not yet been widely applied to clinical trials. Historically, most of the work on gut microbiomes has focused on the dominant bacterial communities, which outpaces that of viral and eukaryotic communities (Shivaji, 2017). Cluster #7 is about gut bacterial microbiome alterations in some extra-intestinal diseases, including central nervous system disorders (multiple sclerosis) (Chen et al., 2016), eye diseases (uveitis, keratitis, and retinitis pigmentosa) (Horai et al., 2017; Jayasudha et al., 2018; Chakravarthy et al., 2018a; Chakravarthy et al., 2018b; Kutsyr et al., 2021), and cardiovascular disease (Tang et al., 2017). In the studies on keratitis, the interaction networks between bacterial and fungal microbiomes in patients, regardless of bacterial or fungal keratitis, were demonstrated (Jayasudha et al., 2018; Chakravarthy et al., 2018b).

**Table 9:** Cited references and citing articles of cluster #5 fecal transplant and #7 bacterial microbiome.

Clusters	Cited references		Citing articles	
	Author (year) journal, volume	Citation counts	Author (year) title	Coverage counts
#5 Fecal transplant				
	Nakamura YK (2017) Sci Rep, 7	29	Choi RY (2018) Fecal transplants in spondyloarthritis and uveitis: ready for a clinical trial?	17
	Lin P (2014) *PLoS One*, 9	13	Rosenbaum JT (2018) The microbiome and HLA-B27-associated acute anterior uveitis	15
	Costello ME (2015) Arthritis Rheumatol, 67	13	Pedersen SJ (2019) The pathogenesis of ankylosing spondylitis: an update	6
#7 Bacterial microbiome				
	Horai R (2017) Expert Rev Clin Immu, 13	9	Jayasudha R (2018) Alterations in gut bacterial and fungal microbiomes are associated with bacterial keratitis, an inflammatory disease of the human eye	16
	Shivaji S (2017) Gut Pathog, 9	8	Chakravarthy SK (2018) Alterations in the gut bacterial microbiome in fungal keratitis patients	13
	Chen J (2016) Sci Rep, 6	5	Chakravarthy SK (2018) Dysbiosis in the gut bacterial microbiome of patients with uveitis, an inflammatory disease of the eye	11
	Tang WHW (2017) Circ Res, 120	5	Kutsyr O (2021) Retinitis pigmentosa is associated with shifts in the gut microbiome	5

## 4 Discussion

In the field of ophthalmic diseases, there is an increasing interest in relieving ocular symptoms by modulating the intestinal commensals, as the commensals play a crucial role in innate and adaptive immunity to achieve favorable control of diseases (Moon et al., 2020c). To the best of our knowledge, this study is the first bibliometric study and visualization analysis about the effects of gut microbiota on ocular disorders.

### 4.1 Brief history of research on gut microbiota in ocular diseases

The first article in this research field is a letter to the editor, published in 2009, about gut microbiota’s influence on the lens and retinal lipid metabolism. Thus, it opened up a new area connecting gut microbiota with ocular health (Oresic et al., 2009).

In the following five years, fewer than ten papers were published, mainly about HLA-B27, one of the main risk factors for ankylosing spondylitis and spondyloarthritis-associated uveitis, affecting the intestinal microbiome of transgenic rats (Lin et al., 2013; Lin et al., 2014), commensal microbiota in the pathophysiology and treatment of irritable bowel, eye and mind syndrome (Feher et al., 2014), and early optimal nutrition improving neurodevelopmental outcomes in infants, including but not limited to retinopathy of prematurity (Hsiao et al., 2014). Notably, the idea of ameliorating EAU by altering the gut microbiota began to be raised, albeit in the form of a conference abstract in 2014 (Nakamura et al., 2014). Meanwhile, terms such as ankylosing spondylitis, inflammatory bowel disease, autoimmune uveitis, and HLA-B27 transgenic rats became burst keywords in 2013 (**Figure 5B**). At this time, the involvement of the microbiota in the eye was just in its infancy.

Subsequently, researches on this topic have grown steadily since 2016 (**Figure 2A**), just after the first report on the microbiota-dependent activation of autoimmunity in the mouse model of spontaneous uveitis in 2015 (Horai et al., 2015). In the years 2016 and 2017, the involvement of diet, gut microbiota, or microbial metabolites such as short-chain fatty acids in immune-mediated uveitis (Heissigerova et al., 2016; Nakamura et al., 2016; Nakamura et al., 2017), Sjogren’s syndrome (de Paiva et al., 2016) and AMD (Andriessen et al., 2016; Rowan et al., 2017; Zinkernagel et al., 2017) was gradually emerging. These works are ground-breaking studies in ocular diseases, mainly from the United States, Switzerland, Canada, and the Czech Republic, most of which are also the top co-cited references in **Table 5**. It was not until 2018 that the first data on the effects of intermittent fasting on DR in db/db mice were reported (Beli et al., 2018).

### 4.2 Leading countries, top authors, top co-cited authors, and leading journals

From the perspective of countries, the United States has absolute leadership in this field, with 2306 citations and the highest centrality (**Table 1**), and more than half of the top authors and co-cited authors are from the United States (**Table 2**). This result may reflect the solid financial and institutional support behind it. In second place is the People’s Republic of China, with 1142 citations and a centrality of 0.40, from which Huang XY, one of the productive authors in the area, comes. It is followed by the United Kingdom, Italy, and Japan with similar counts of citations, whereas there is a lower centrality in Japan (**Table 1**). Shivaji S, Huang XY, and Kim MK are three authors from Asia on the list of top authors, yet there are no Asian authors in the top co-cited author list (**Table 2**).

As for the top authors and top co-cited authors, there is a high degree of overlapping (**Table 2**). Lin P is the most productive author whose significant contributions lie in the studies of modifying the gut microbiota to prevent EAU (Nakamura et al., 2016; Nakamura et al., 2017; Janowitz et al., 2019) and exploring the potential role of HLA-B27 in the process (Lin et al., 2013; Lin et al., 2014), as well as follow-up reviews about the role of the gut microbiome in AMD, another ocular inflammatory disease (Lin, 2018; Lin, 2019; Lin et al., 2021). Moreover, Lin P, Rosenbaum JT, Asquith M, and Nakamura YK have a close cooperative relationship; they are all scholars from the same university, Oregon Health and Science University. Except for Asquith M, they are also the top co-cited authors on this topic (**Table 2**). Similarly, Horai R and Caspi RR are professors from the same laboratory and work on the pathogenesis of commensal microbiota in the model of spontaneous uveitis (Horai et al., 2015; Zarate-Blades et al., 2017). At the same time, Horai R is the first co-cited author in **Table 2**. Considering the distribution of authors with their research direction, it can be inferred that studies on uveitis are relatively mature, with the most significant number in the whole subject.

In terms of published journals, *IOVS*, one of the most influential ophthalmology journals, published the most papers, followed by *Scientific Reports* and *Frontiers in Immunology*. Among these co-cited journals, *IOVS* is also the most cited, followed by *Plos One* and *Nature*, which are well-known comprehensive journals (**Table 3**). The studies from these journals broaden and deepen the perception of the interaction between the eye and the gut and provide the possibility for applying the therapeutic strategy, which targets gut microbiota, to clinical scenarios.

### 4.3 Keywords analysis

Co-occurrence and burst analyses of keywords could offer insight into research conditions, hotspots of different directions, and the evolution of frontiers in this area. In our study, co-occurrence networks are broadly consistent with the trends shown by the analysis of burst terms (**Figures 5A, B**). The concern for the intestinal flora stemmed from its performance in inflammatory bowel disease and ankylosing spondylitis (Thompson-Chagoyán et al., 2005; Scanlan et al., 2006; Lin et al., 2014; Costello et al., 2015). Autoimmune uveitis was gradually becoming another focal point, presumably because it is the most common ocular manifestation of systemic immune diseases (Rosenbaum and Kim, 2013; Consolandi et al., 2015; Thomas and Lin, 2016; Sharma and Jackson, 2017). In these uveitis-related studies, the hypothesis and discovery by Horai R et al. (Horai et al., 2015) and Nakamura YK et al. (Nakamura et al., 2016) regarding the involvement of gut microbiota in the pathogenic mechanism have given considerable impetus to the advancement of this field. From this, a research boom was set off in the “gut-eye” axis research. Since bacteria are the prominent and most known component of the gut microbiota, the frequency of bacteria and diversity (including bacterial α diversity and β diversity) also exploded in a period. Subsequently, regulatory T cells became a high-frequency keyword because commensal microbes mainly regulate intestinal and parenteral immunity by balancing regulatory T cells and Th17 cells, and Th17 cells can induce inflammatory responses while regulatory T cells are essential in suppressing excessive inflammation (Nakamura et al., 2016; Nakamura et al., 2017; Rosenbaum and Asquith, 2018; Zamvil et al., 2018). The role of microbiota in maintaining ocular surface health and barrier, as well as preventing immune-mediated ocular surface diseases such as dry eye manifestations caused by Sjogren’s syndrome, also began to be valued (**Figure 5B**) (de Paiva et al., 2016; Kugadas et al., 2017).

### 4.4 Graves’ orbitopathy (GO) mouse model paper has the highest betweenness centrality

In the analysis of co-cited references, “betweenness centrality” is the ability of every reference to mediate between other papers in an interaction network. The greater the betweenness centrality, the stronger the ability to connect different sections (Lin et al., 2022). Surprisingly, while the number of documents on gut dysbiosis and GO is limited, the paper with the highest betweenness centrality among all the co-cited references comes from cluster #6 (graves’ orbitopathy) (**Table 6**) (Berchner-Pfannschmidt et al., 2016). This document established a preclinical model of experimental GO in female BALB/c mice and evaluated changes in pro-inflammatory cytokines and T cell immune responses at two research centers. Although alterations in the intestine were not mentioned, the establishment of this animal model undoubtedly provides excellent convenience and solid supports for the study of the underlying mechanism of GO, including the association with the intestine microbiota. It is the probable reason why it owns the highest betweenness centrality. Indeed, the subsequent study used this mouse model to see the correlation of gut microbiota changes with the clinical presentation of GO under different environments (Masetti et al., 2018). Most of the other literature with high betweenness centrality comes from clusters #1 (autoinflammatory uveitis) and #3 (microbiome-linked control) about uveitis or regulatory T cells (Arnold et al., 2011; Atarashi et al., 2013; Horai et al., 2015; Nakamura et al., 2016; Avni and Koren, 2018; Huang et al., 2018).

### 4.5 The mechanisms of gut dysbiosis in the pathogenesis of autoimmune uveitis

Moreover, cluster analysis divides co-cited references into several typical clusters (**Figure 6A**). For autoimmune diseases and autoinflammatory diseases, there is a relatively vague distinction. Autoimmune diseases occur when the adaptive immune system’s immune tolerance to autoantigens is disrupted, while autoinflammatory diseases develop when the innate immune system is defective or dysregulated (Ben-Chetrit et al., 2018; Molzer et al., 2021). Classic autoimmune diseases include multiple sclerosis (Choileain et al., 2020), type 1 diabetes (Dedrick et al., 2020), and rheumatoid arthritis (Manasson et al., 2020), while inflammatory bowel disease and ankylosing spondylitis are classified as probable autoinflammatory diseases (Vural et al., 2020). In many cases, uveitis, whose cause can be direct or indirect, is regarded as either an autoimmune or autoinflammatory disease (Forrester et al., 2018; Molzer et al., 2021). Thus, although cluster #1 is named “autoinflammatory uveitis” in the map, many cited papers in this cluster and our study do not make a strict distinction between the two categories. As repeatedly emphasized before, uveitis is the first ocular abnormality found to be associated with gut microbiota, including EAU mouse models and acute anterior uveitis patients (Huang et al., 2018), and it plays a pivotal role in mechanistic research. Clusters #1, #3, and the timeline map also keep verifying this notion (**Figure 6B**).

Various possible mechanisms mediated by intestinal dysbiosis have been proposed autoimmune uveitis. First, bacteria can regulate the balance of Th17 cells and regulatory T cells in the gut, leading to loss of immune homeostasis (Zhuang et al., 2017). Increased Th17 cell and decreased regulatory T cells predispose to immune-mediated diseases. For instance, a variety of *Klebsiella* strains were reported to promote the production of Th cells in the gut of mice (Atarashi et al., 2017). Second, intestinal bacterial antigens can induce cross-reaction by mimicking autoantigens, thus activating adaptive immune responses (Avni and Koren, 2018). In the spontaneously uveitic R161H mice model, the gut commensal microbiota is possible to activate retina-specific T cells by mimicking IRBP to cause disease (Horai et al., 2015). The presence of cross-reactivity of microorganisms is also confirmed in systemic lupus erythematosus. *Propionibacterium propionicum* and *Bacteroides thetaiotaomicron*, two of the identified commensal microorganisms, were regarded to activate Ro60-specific CD4^+^ memory T cells in lupus patients (Avni and Koren, 2018; Greiling et al., 2018). Third, dysbiosis of gut microbiota may cause the alteration in intestinal permeability. This change in permeability allows some bacterial products (such as lipopolysaccharides, β-glucans) to spread into blood vessels and tissues and stay in tissues like synovium or uvea, which could trigger the immune response to induce arthritis or uveitis (Rosenbaum and Asquith, 2018; Parthasarathy et al., 2023). Finally, promoting the migration of immune cells to extra-intestinal regions may also be one of the critical pathogenic mechanisms (Morton et al., 2014). It was found that at the peak of inflammation (about two weeks after immunization) in EAU model, the pathogenic bacteria, represented by *Prevotella*, increased significantly, accompanied by an increase in the transportation of leukocytes between the intestine and the eye (Nakamura et al., 2017).

### 4.6 Therapeutic strategies targeting gut microbiota to treat ocular diseases

Concomitantly, in both DR (cluster #0) and AMD (cluster #2) studies, the positive effects of diet on disease control have been described, whether the modifications in dietary style (intermittent fasting) or improvements in dietary patterns (high-fat diet and high-glycemia diet) (Andriessen et al., 2016; Rowan et al., 2017; Beli et al., 2018). Oral probiotics like IRT5 have been reported to have a certain effect in experimental dry eye (cluster #4) models by changing microbiota composition (Kim et al., 2017; Choi et al., 2020; Moon et al., 2020b). These suggest that dietary modification and oral probiotics are significant ways to treat and alleviate gut microbiota-related diseases. GD- and GO-related (cluster #6) studies started late in this area, in which more animal experiments and clinical trials are needed.

In addition to dietary modifications and the use of probiotics, FMT (cluster #5) is another method of manipulating the microbiota and has been reported to be effective in the treatment of colitis caused by recurrent *Clostridium difficile* infection (van Nood et al., 2013; Cheng et al., 2019). Several review articles have also described and looked forward to the potential effectiveness of FMT for the treatment of extra-intestinal diseases, including ocular disorders (Choi et al., 2018; Rosenbaum and Asquith, 2018; Baim et al., 2019; Fu et al., 2021; Hou et al., 2021). Specially, a recent clinical trial published in the *American Journal of Ophthalmology* used FMT to treat dry eye patients. 10 recipients received two FMTs from a single healthy donor *via* enema (Watane et al., 2022). Despite being limited by the small sample size, only short-term microbial composition close to the donor, and subjective reported symptom improvement, this study undoubtedly promoted advances in FMT for autoimmune eye diseases. Moreover, some studies transplanted the feces of patients into germ-free or antibiotic-treated mouse models of the corresponding disease, and found that this exacerbated their current disease manifestations (Andriessen et al., 2016; Su et al., 2020; Ye et al., 2020; Schaefer et al., 2022b), indirectly demonstrating the role of intestinal dysbiosis in the pathogenesis. The application of FMT still needs to be improved, and there is a long way to go before FMT can be generally applied in clinical treatment.

Apart from gut microbiota itself, microbial metabolites are also an important target. Short-chain fatty acids (SCFAs) are the most commonly found beneficial bacterial metabolites, including acetic acid, propionic acid, and butyric acid. A recent study showed that fenofibrate, a lipid-lowering drug, can reduce retinal inflammation in high-fat diet-induced mice and reverse the decline of SCFAs in serum, retina, and feces (Wang et al., 2022). At the same time, the number of lipopolysaccharide-associated bacteria was reduced, such as *Desulfovibrionaceae* family, *Acetatifactor*, *Flavonifractor*, *Oscillibacter*, and *Anaerotruncus* genus, while SCFA-associated bacteria increased, including *Porphyromonadaceae* family, *Barnesiella*, *Alloprevotella*, and *Bifidobacterium* genus (Wang et al., 2022). Likewise, intra-peritoneal injected SCFAs can be detected in the eye by crossing the blood-eye barrier and inhibiting lipopolysaccharide-induced intraocular inflammation (Chen et al., 2021). Oral propionic acid has been reported to inhibit the migration of gut-spleen effector T cells and prevent the transport of leukocytes between the intestine and extra-intestinal tissues, thereby alleviating the severity of uveitis in EAU mice (Nakamura et al., 2017). Similarly, gut-derived butyrate was proposed to potentially suppress ocular surface inflammation, which is beneficial to the dry eye mouse model (Schaefer et al., 2022a). Besides, in the feces of acute anterior uveitis patients, Huang et al. (Huang et al., 2018) identified seven elevated metabolites which are associated with certain inflammatory or immune-mediated diseases, such as inflammatory bowel disease and non-alcoholic fatty liver disease. Still, the link to the eye needs further discovery. Since commensal microbiota produces thousands of metabolites, our understanding of them must be clarified. Identifying ophthalmic-specific metabolites and the regulatory gut microbes may be one direction of future efforts.

### 4.7 Fungal mycobiome may play a role in ocular diseases

By now, dysbiosis in the bacterial microbiome (cluster #7) is the most studied and described. However, other agents, such as viral and fungal communities, are also resident in the intestine, and their dysregulation can lead to various diseases. However, sequencing studies of fungal microbiota are gradually emerging. Alterations in bacterial and fungal microbiomes, as well as their interaction networks, were analyzed (Jayasudha et al., 2018; Chakravarthy et al., 2018a; Chakravarthy et al., 2018b; Jayasudha et al., 2019). In the future, the gut virus and the fungal microbiota deserve more findings to improve our knowledge and understanding of gut dysbiosis.

Overall, based on bibliometric methods, by integrating nearly 15 years of relevant literature, our study displays the research process, research hotspots, and developmental research directions of the involvement of gut microbiota in the pathogenesis and treatment of ocular diseases and provides an overview of the dynamic evolution and structural relationships in the field. The link between the eye and commensals in the gut has further significance and value. With the addition of more high-quality results, future research on microbiota and eye disorders will be conducted continuously and dynamically.

### 4.8 Limitations of this study

There are some limitations in our study. (1) All publications we included come only from the WoS Core Collection database, and other commonly used databases, such as Scopus, PubMed, Embase, and Medline, may help provide more comprehensive literature coverage. (2) The study of intestinal microbiota is an emerging field, so the number of articles we have retrieved and the corresponding time still need to be improved, and the analysis and prediction of trends and hotspots also need to undergo a more extended period of verification. (3)Our bibliometric study and visualization analysis mainly rely on two software, CiteSpace, and VOSviewer. The software algorithm sometimes has some deviations. For example, in the cluster analysis, references from clusters #1 and #3 have a strong correlation in content, and it can be seen that the documents of the two clusters are continuous on the timeline. But in the cluster diagram, these documents are divided into two non-overlapping parts (**Figures 6A, B**).

## 5 Conclusion

We are amidst an explosion of research on gut microbiota in ocular diseases. The United States, where most prominent authors come from, is leading the way in this field. Papers or abstracts published in the ophthalmology journal IOVS, receive the most attention. Intestinal dysbiosis is involved in various common immune- and inflammation-mediated ocular diseases, including but not limited to uveitis, diabetic retinopathy, age-related macular degeneration, dry eye, and Graves’ orbitopathy. With the deepening of the understanding of gut microbiota, other eye diseases, such as glaucoma, retinopathy of prematurity, retinitis pigmentosa and retinal artery occlusion, on which related studies are still limited. Indeed, the relationship between these ocular diseases and gut dysbiosis needs to be further investigated.

Meanwhile, the study of microbiomes is no longer limited to bacterial populations. Several studies suggested that the gut fungal mycobiome may be involved in the development of uveitis and keratitis. Even though fungi are much less than bacteria in the gut, commensal fungi have essential roles in human health and disease, and their role in ocular diseases should be carefully explored. In terms of the therapeutic strategies that target the gut microbiota, including probiotics, healthy diet patterns, FMT, and SCFAs, these data are mainly from experimental animal models, and there is a need for more human-based clinical trials to determine their efficacy in clinical settings.

## Data availability statement

The original contributions presented in the study are included in the article/supplementary material. Further inquiries can be directed to the corresponding authors.

## Author contributions

XF and DC designed the study and wrote the first draft of the manuscript. XF, HT, LH, WC, XR, and DC revised the manuscript. All authors participated in the literature search and data analysis, and have read and approved the final manuscript.

## References

[B1] Andriessen E. Wilson A. M. Mawambo G. Dejda A. Miloudi K. Sennlaub F.. (2016). Gut microbiota influences pathological angiogenesis in obesity-driven choroidal neovascularization. EMBO Mol. Med. 8, 1366–1379. doi: 10.15252/emmm.201606531 27861126 PMC5167134

[B2] Arnold I. C. Dehzad N. Reuter S. Martin H. Becher B. Taube C.. (2011). Helicobacter pylori infection prevents allergic asthma in mouse models through the induction of regulatory T cells. J. Clin. Invest. 121, 3088–3093. doi: 10.1172/JCI45041 21737881 PMC3148731

[B3] Atarashi K. Suda W. Luo C. Kawaguchi T. Motoo I. Narushima S.. (2017). Ectopic colonization of oral bacteria in the intestine drives T(H)1 cell induction and inflammation. Science 358, 359–365. doi: 10.1126/science.aan4526 29051379 PMC5682622

[B4] Atarashi K. Tanoue T. Oshima K. Suda W. Nagano Y. Nishikawa H.. (2013). Treg induction by a rationally selected mixture of Clostridia strains from the human microbiota. Nature 500, 232–236. doi: 10.1038/nature12331 23842501

[B5] Avni O. Koren O. (2018). Molecular (Me)micry? Cell Host Microbe 23, 576–578. doi: 10.1016/j.chom.2018.04.012 29746828

[B6] Bai W. H. Gu D. F. Dai Y. Chen Y. H. Yang Z. M. Lu L. J. (2023a). The relationship between probiotics and retinopathy of prematurity in preterm infants: A population-based retrospective study in China. Front. IN Pediatr. 11, 1055992. doi: 10.3389/fped.2023.1055992 PMC9989163 36896406

[B7] Bai X. D. Xu Q. Zhang W. N. Wang C. Y. (2023b). The gut-eye axis: correlation between the gut microbiota and autoimmune dry eye in individuals with Sjogren syndrome. EYE CONTACT LENS-SCIENCE AND Clin. Pract. 49, 1–7. doi: 10.1097/ICL.0000000000000953 36544282

[B8] Baim A. D. Movahedan A. Farooq A. V. Skondra D. (2019). The microbiome and ophthalmic disease. Exp. Biol. AND Med. 244, 419–429. doi: 10.1177/1535370218813616 PMC6546998 30463439

[B9] Beli E. Yan Y. Q. Moldovan L. Vieira C. P. Gao R. Duan Y. Q.. (2018). Restructuring of the gut microbiome by intermittent fasting prevents retinopathy and prolongs survival in db/db mice. DIABETES 67, 1867–1879. doi: 10.2337/db18-0158 29712667 PMC6110320

[B10] Ben-Chetrit E. Gattorno M. Gul A. Kastner D. L. Lachmann H. J. Touitou I.. (2018). Consensus proposal for taxonomy and definition of the autoinflammatory diseases (AIDs): a Delphi study. Ann. Rheum Dis. 77, 1558–1565. doi: 10.1136/annrheumdis-2017-212515 30100561

[B11] Berchner-Pfannschmidt U. Moshkelgosha S. Diaz-Cano S. Edelmann B. Gortz G. E. Horstmann M.. (2016). Comparative assessment of female mouse model of graves’ Orbitopathy under different environments, accompanied by proinflammatory cytokine and T-cell responses to thyrotropin hormone receptor antigen. Endocrinology 157, 1673–1682. doi: 10.1210/en.2015-1829 26872090

[B12] Berer K. Mues M. Koutrolos M. Rasbi Z. A. Boziki M. Johner C.. (2011). Commensal microbiota and myelin autoantigen cooperate to trigger autoimmune demyelination. Nature 479, 538–541. doi: 10.1038/nature10554 22031325

[B13] Biscarini F. Masetti G. Muller I. Verhasselt H. L. Covelli D. Colucci G.. (2023). Gut microbiome associated with graves disease and graves orbitopathy: the INDIGO multicenter European study. J. Clin. Endocrinol. Metab 108 (8), 2065–2077. doi: 10.1210/clinem/dgad030 PMC10807910 36683389

[B14] Brunkwall L. Orho-Melander M. (2017). The gut microbiome as a target for prevention and treatment of hyperglycaemia in type 2 diabetes: from current human evidence to future possibilities. Diabetologia 60, 943–951. doi: 10.1007/s00125-017-4278-3 28434033 PMC5423958

[B15] Cao Y. Q. Li Y. L. Gkerdi A. Reilly J. Tan Z. J. Shu X. H. (2022). Association of nutrients, specific dietary patterns, and probiotics with age-related macular degeneration. Curr. MEDICINAL Chem. 29, 6141–6158. doi: 10.2174/0929867329666220511142817 35546762

[B16] Cavuoto K. M. Banerjee S. Galor A. (2019). Relationship between the microbiome and ocular health. OCULAR SURFACE 17, 384–392. doi: 10.1016/j.jtos.2019.05.006 31125783

[B17] Chakravarthy S. K. Jayasudha R. Prashanthi G. S. Ali M. H. Sharma S. Tyagi M.. (2018a). Dysbiosis in the gut bacterial microbiome of patients with uveitis, an inflammatory disease of the eye. Indian J. OF Microbiol. 58, 457–469. doi: 10.1007/s12088-018-0746-9 30262956 PMC6141402

[B18] Chakravarthy S. K. Jayasudha R. Ranjith K. Dutta A. Pinna N. K. Mande S. S.. (2018b). Alterations in the gut bacterial microbiome in fungal Keratitis patients. PLoS One 13, e0199640. doi: 10.1371/journal.pone.0199640 29933394 PMC6014669

[B19] Chen C. (2006). CiteSpace II: Detecting and visualizing emerging trends and transient patterns in scientific literature. J. Am. Soc. Inf. Sci. Technol. 57, 359–377. doi: 10.1002/asi.20317

[B20] Chen J. Chia N. Kalari K. R. Yao J. Z. Novotna M. Paz Soldan M. M.. (2016). Multiple sclerosis patients have a distinct gut microbiota compared to healthy controls. Sci. Rep. 6, 28484. doi: 10.1038/srep28484 27346372 PMC4921909

[B21] Chen C. M. Dubin R. Kim M. C. (2014). Emerging trends and new developments in regenerative medicine: a scientometric update, (2000-2014). Expert Opin. Biol. Ther. 14, 1295–1317. doi: 10.1517/14712598.2014.920813 25077605

[B22] Chen N. Wu J. Wang J. R. Piri N. Chen F. L. Xiao T.. (2021). Short chain fatty acids inhibit endotoxin-induced uveitis and inflammatory responses of retinal astrocytes. Exp. EYE Res. 206, 108520. doi: 10.1016/j.exer.2021.108520 33617852 PMC8489808

[B23] Cheng Y. W. Phelps E. Ganapini V. Khan N. Ouyang F. Xu H.. (2019). Fecal microbiota transplantation for the treatment of recurrent and severe Clostridium difficile infection in solid organ transplant recipients: A multicenter experience. Am. J. Transplant. 19, 501–511. doi: 10.1111/ajt.15058 30085388 PMC6349556

[B24] Choi R. Y. Asquith M. Rosenbaum J. T. (2018). Fecal transplants in spondyloarthritis and uveitis: ready for a clinical trial? Curr. Opin. IN Rheumatol. 30, 303–309. doi: 10.1097/BOR.0000000000000506 29538010

[B25] Choi S. H. Oh J. W. Ryu J. S. Kim H. M. Im S. H. Kim K. P.. (2020). IRT5 probiotics changes immune modulatory protein expression in the extraorbital lacrimal glands of an autoimmune dry eye mouse model. Invest. Ophthalmol. Visual Sci. 61, 42. doi: 10.1167/iovs.61.3.42 PMC7401425 32232342

[B26] Choileain S. N. Kleinewietfeld M. Raddassi K. Hafler D. A. Ruff W. E. Longbrake E. E. (2020). CXCR3+ T cells in multiple sclerosis correlate with reduced diversity of the gut microbiome. J. Transl. Autoimmun 3, 100032. doi: 10.1016/j.jtauto.2019.100032 32743517 PMC7388357

[B27] Ciccia F. Guggino G. Rizzo A. Alessandro R. Luchetti M. M. Milling S.. (2017). Dysbiosis and zonulin upregulation alter gut epithelial and vascular barriers in patients with ankylosing spondylitis. Ann. Rheum Dis. 76, 1123–1132. doi: 10.1136/annrheumdis-2016-210000 28069576 PMC6599509

[B28] Consolandi C. Turroni S. Emmi G. Severgnini M. Fiori J. Peano C.. (2015). Behcet’s syndrome patients exhibit specific microbiome signature. Autoimmun. Rev. 14, 269–276. doi: 10.1016/j.autrev.2014.11.009 25435420

[B29] Costello M. E. Ciccia F. Willner D. Warrington N. Robinson P. C. Gardiner B.. (2015). Brief report: intestinal dysbiosis in ankylosing spondylitis. Arthritis Rheumatol 67, 686–691. doi: 10.1002/art.38967 25417597

[B30] Das T. Jayasudha R. Chakravarthy S. Prashanthi G. S. Bhargava A. Tyagi M.. (2021). Alterations in the gut bacterial microbiome in people with type 2 diabetes mellitus and diabetic retinopathy. Sci. Rep. 11, 2738. doi: 10.1038/s41598-021-82538-0 33531650 PMC7854632

[B31] Dedrick S. Sundaresh B. Huang Q. Brady C. Yoo T. Cronin C.. (2020). The role of gut microbiota and environmental factors in type 1 diabetes pathogenesis. Front. Endocrinol. (Lausanne) 11, 78. doi: 10.3389/fendo.2020.00078 32174888 PMC7057241

[B32] de Paiva C. S. Jones D. B. Stern M. E. Bian F. Moore Q. L. Corbiere S.. (2016). Altered mucosal microbiome diversity and disease severity in Sjogren syndrome. Sci. Rep. 6, 23561. doi: 10.1038/srep23561 27087247 PMC4834578

[B33] Diez-Sainz E. Lorente-Cebrian S. Aranaz P. Riezu-Boj J. I. Martinez J. A. Milagro F. I. (2021). Potential mechanisms linking food-derived microRNAs, gut microbiota and intestinal barrier functions in the context of nutrition and human health. Front. Nutr. 8, 586564. doi: 10.3389/fnut.2021.586564 33768107 PMC7985180

[B34] Fang J. Yu C. H. Li X. J. Yao J. M. Fang Z. Y. Yoon S. H.. (2022). Gut dysbiosis in nonalcoholic fatty liver disease: pathogenesis, diagnosis, and therapeutic implications. Front. Cell Infect. Microbiol. 12, 997018. doi: 10.3389/fcimb.2022.997018 36425787 PMC9679376

[B35] Feher J. Kovacs I. Elena P. Radak Z. (2014). Microbiota-host symbiosis in the pathophysiology and treatment of irritable bowel, irritable eye and irritable mind syndrome. ORVOSI HETILAP 155, 1454–1460. doi: 10.1556/OH.2014.29987 25194867

[B36] Forrester J. V. Kuffova L. Dick A. D. (2018). Autoimmunity, autoinflammation, and infection in uveitis. Am. J. Ophthalmol. 189, 77–85. doi: 10.1016/j.ajo.2018.02.019 29505775

[B37] Fu X. Y. Chen Y. J. Chen D. N. (2021). The role of gut microbiome in autoimmune uveitis. OPHTHALMIC Res. 64, 168–177. doi: 10.1159/000510212 32674100

[B38] Gianchecchi E. Fierabracci A. (2019). Recent advances on microbiota involvement in the pathogenesis of autoimmunity. Int. J. Mol. Sci. 20 (2), 283. doi: 10.3390/ijms20020283 PMC6359510 30642013

[B39] Greiling T. M. Dehner C. Chen X. Hughes K. Iñiguez A. J. Boccitto M.. (2018). Commensal orthologs of the human autoantigen Ro60 as triggers of autoimmunity in lupus. Sci. Transl. Med. 10 (434), eaan2306. doi: 10.1126/scitranslmed.aan2306 PMC5918293 29593104

[B40] Gritz E. C. Bhandari V. (2015). The human neonatal gut microbiome: a brief review. Front. Pediatr. 3, 17. doi: 10.3389/fped.2015.00017 25798435 PMC4350424

[B41] Gu X. Xie M. Y. Jia R. B. Ge S. F. (2021). Publication trends of research on retinoblastoma during 2001-2021: A 20-year bibliometric analysis. Front. Med. 8. doi: 10.3389/fmed.2021.675703 PMC8175655 34095180

[B42] Guler A. T. Waaijer C. J. F. Palmblad M. (2016). Scientific workflows for bibliometrics. Scientometrics 107, 385–398. doi: 10.1007/s11192-016-1885-6 27122644 PMC4833826

[B43] Heissigerova J. Stangova P. S. Klimova A. Svozilkova P. Hrncir T. Stepankova R.. (2016). The microbiota determines susceptibility to experimental autoimmune uveoretinitis. J. OF Immunol. Res. 2016, 5065703. doi: 10.1155/2016/5065703 27294159 PMC4886056

[B44] Horai R. Caspi R. R. (2019). Microbiome and autoimmune uveitis. Front. IN Immunol. 10, 232. doi: 10.3389/fimmu.2019.00232 30837991 PMC6389708

[B45] Horai R. Sen H. N. Caspi R. R. (2017). Commensal microbiota as a potential trigger of autoimmune uveitis. Expert Rev. Clin. Immunol. 13, 291–293. doi: 10.1080/1744666X.2017.1288098 28145784 PMC5546913

[B46] Horai R. Zarate-Blades C. R. Dillenburg-Pilla P. Chen J. Kielczewski J. L. Silver P. B.. (2015). Microbiota-dependent activation of an autoreactive T cell receptor provokes autoimmunity in an immunologically privileged site. IMMUNITY 43, 343–353. doi: 10.1016/j.immuni.2015.07.014 26287682 PMC4544742

[B47] Hou J. Y. Tang Y. J. Chen Y. J. Chen D. N. (2021). The role of the microbiota in Graves’ Disease and Graves’ Orbitopathy. Front. IN Cell. AND INFECTION Microbiol. 11, 739707. doi: 10.3389/fcimb.2021.739707 PMC8727912 35004341

[B48] Hsiao C. C. Tsai M. L. Chen C. C. Lin H. C. (2014). Early optimal nutrition improves neurodevelopmental outcomes for very preterm infants. Nutr. Rev. 72, 532–540. doi: 10.1111/nure.12110 24938866

[B49] Hu X. Wang T. Jin F. (2016). Alzheimer’s disease and gut microbiota. Sci. China Life Sci. 59, 1006–1023. doi: 10.1007/s11427-016-5083-9 27566465

[B50] Huang X. Y. Ye Z. Cao Q. F. Su G. N. Wang Q. F. Deng J.. (2018). Gut microbiota composition and fecal metabolic phenotype in patients with acute anterior uveitis. Invest. Ophthalmol. Visual Sci. 59, 1523–1531. doi: 10.1167/iovs.17-22677 29625474

[B51] Ishaq H. M. Mohammad I. S. Shahzad M. Ma C. F. Raza M. A. Wu X. K.. (2018). Molecular alteration analysis of human gut microbial composition in Graves’ disease patients. Int. J. OF Biol. Sci. 14, 1558–1570. doi: 10.7150/ijbs.24151 30263008 PMC6158725

[B52] Janowitz C. Nakamura Y. K. Metea C. Gligor A. Yu W. Karstens L.. (2019). Disruption of intestinal homeostasis and intestinal microbiota during experimental autoimmune uveitis. Invest. Ophthalmol. Visual Sci. 60, 420–429. doi: 10.1167/iovs.18-24813 30695094 PMC6353239

[B53] Jayasudha R. Chakravarthy S. K. Prashanthi G. S. Sharma S. Garg P. Murthy S. I.. (2018). Alterations in gut bacterial and fungal microbiomes are associated with bacterial Keratitis, an inflammatory disease of the human eye. J. OF Biosci. 43, 835–856. doi: 10.1007/s12038-018-9798-6 30541945

[B54] Jayasudha R. Chakravarthy S. K. Prashanthi G. S. Sharma S. Tyagi M. Shivaji S. (2019). Implicating dysbiosis of the gut fungal microbiome in uveitis, an inflammatory disease of the eye. Invest. Ophthalmol. Visual Sci. 60, 1384–1393. doi: 10.1167/iovs.18-26426 30938773

[B55] Jiao J. H. Yu H. H. Yao L. T. Li L. H. Yang X. H. Liu L. (2021). Recent insights into the role of gut microbiota in diabetic retinopathy. J. Inflammation Res. 14, 6929–6938. doi: 10.2147/JIR.S336148 PMC8687677 34938095

[B56] Kim J. Choi S. H. Kim Y. J. Jeong H. J. Ryu J. S. Lee H. J.. (2017). Clinical effect of IRT-5 probiotics on immune modulation of autoimmunity or alloimmunity in the eye. Nutrients 9 (11), 1166. doi: 10.3390/nu9111166 PMC5707638 29068389

[B57] Kodati S. Sen H. N. (2019). Uveitis and the gut microbiota. Best Pract. Res. Clin. Rheumatol. 33, 101500. doi: 10.1016/j.berh.2020.101500 32278666 PMC7299813

[B58] Kugadas A. Wright Q. Geddes-Mcalister J. Gadjeva M. (2017). Role of microbiota in strengthening ocular mucosal barrier function through secretory IgA. Invest. Ophthalmol. Visual Sci. 58, 4593–4600. doi: 10.1167/iovs.17-22119 28892827 PMC5595225

[B59] Kumar K. Dhoke G. V. Sharma A. K. Jaiswal S. K. Sharma V. K. (2019). Mechanistic elucidation of amphetamine metabolism by tyramine oxidase from human gut microbiota using molecular dynamics simulations. J. Cell Biochem. 120, 11206–11215. doi: 10.1002/jcb.28396 30701587

[B60] Li Y. Luo B. Tong B. Xie Z. Cao J. Bai X.. (2023). The role and molecular mechanism of gut microbiota in Graves’ orbitopathy. J. ENDOCRINOL. Invest. 46, 305–317. doi: 10.1007/s40618-022-01902-7 35986869

[B61] Lima-Fontes M. Meira L. Barata P. Falcao M. Carneiro A. (2022). Gut microbiota and age-related macular degeneration: A growing partnership. SURVEY OF Ophthalmol. 67, 883–891. doi: 10.1016/j.survophthal.2021.11.009 34843745

[B62] Lin P. (2018). The role of the intestinal microbiome in ocular inflammatory disease. Curr. Opin. IN Ophthalmol. 29, 261–266. doi: 10.1097/ICU.0000000000000465 29538183

[B63] Lin P. (2019). Importance of the intestinal microbiota in ocular inflammatory diseases: A review. Clin. AND Exp. Ophthalmol. 47, 418–422. doi: 10.1111/ceo.13493 30834680

[B64] Lin P. Bach M. Asquith M. Lee A. Y. Akileswaran L. Stauffer P.. (2014). HLA-B27 and human beta 2-microglobulin affect the gut microbiota of transgenic rats. PLoS One 9, e105684. doi: 10.1371/journal.pone.0105684 25140823 PMC4139385

[B65] Lin P. Bach M. Lee A. Akileswaran L. Taurog J. Rosenbaum J.. (2013). HLA-B27 affects the gut microbiome of transgenic rats. Invest. Ophthalmol. Visual Sci. 54.

[B66] Lin P. Mcclintic S. M. Nadeem U. Skondra D. (2021). A review of the role of the intestinal microbiota in age-related macular degeneration. J. Clin. Med. 10 (10), 2072. doi: 10.3390/jcm10102072 34065988 PMC8151249

[B67] Lin Y. Ren X. Chen D. (2022). Steroid treatment in macular edema: A bibliometric study and visualization analysis. Front. Pharmacol. 13, 824790. doi: 10.3389/fphar.2022.824790 35273502 PMC8902303

[B68] Manasson J. Blank R. B. Scher J. U. (2020). The microbiome in rheumatology: Where are we and where should we go? Ann. Rheumatic Dis. 79, 727–733. doi: 10.1136/annrheumdis-2019-216631 32332073

[B69] Masetti G. Moshkelgosha S. Kohling H. L. Covelli D. Banga J. P. Berchner-Pfannschmidt U.. (2018). Gut microbiota in experimental murine model of Graves’ orbitopathy established in different environments may modulate clinical presentation of disease. MICROBIOME 6, 97. doi: 10.1186/s40168-018-0478-4 29801507 PMC5970527

[B70] Molzer C. Wilson H. M. Kuffova L. Forrester J. V. (2021). A role for folate in microbiome-linked control of autoimmunity. J. Immunol. Res. 2021, 9998200. doi: 10.1155/2021/9998200 34104654 PMC8159645

[B71] Moon J. Choi S. H. Yoon C. H. Kim M. K. (2020a). Gut dysbiosis is prevailing in Sjogren’s syndrome and is related to dry eye severity. PLoS One 15, e0229029. doi: 10.1371/journal.pone.0229029 32059038 PMC7021297

[B72] Moon J. Ryu J. S. Kim J. Y. Im S. H. Kim M. K. (2020b). Effect of IRT5 probiotics on dry eye in the experimental dry eye mouse model. PLoS One 15, e0243176. doi: 10.1371/journal.pone.0243176 33259525 PMC7707591

[B73] Moon J. Yoon C. H. Choi S. H. Kim M. K. (2020c). Can gut microbiota affect dry eye syndrome? Int. J. Mol. Sci. 21 (22), 8443. doi: 10.3390/ijms21228443 33182758 PMC7697210

[B74] Morton A. M. Sefik E. Upadhyay R. Weissleder R. Benoist C. Mathis D. (2014). Endoscopic photoconversion reveals unexpectedly broad leukocyte trafficking to and from the gut. Proc. Natl. Acad. Sci. U.S.A. 111, 6696–6701. doi: 10.1073/pnas.1405634111 24753589 PMC4020091

[B75] Nadeem U. Boachie-Mensah M. Zhang J. Skondra D. (2022). Gut microbiome and retinal diseases: an updated review. Curr. Opin. IN Ophthalmol. 33, 195–201. doi: 10.1097/ICU.0000000000000836 35132003

[B76] Nakamura Y. K. Janowitz C. Metea C. Asquith M. Karstens L. Rosenbaum J. T.. (2017). Short chain fatty acids ameliorate immune-mediated uveitis partially by altering migration of lymphocytes from the intestine. Sci. Rep. 7, 11745. doi: 10.1038/s41598-017-12163-3 28924192 PMC5603543

[B77] Nakamura Y. Metea C. Gruner H. Asquith M. Planck S. R. Rosenbaum J. T.. (2014). Altering the gut microbiota ameliorates experimental autoimmune uveitis. Invest. Ophthalmol. Visual Sci. 55.

[B78] Nakamura Y. K. Metea C. Karstens L. Asquith M. Gruner H. Moscibrocki C.. (2016). Gut microbial alterations associated with protection from autoimmune uveitis. Invest. Ophthalmol. Visual Sci. 57, 3747–3758. doi: 10.1167/iovs.16-19733 27415793 PMC4960998

[B79] Oresic M. Seppanen-Laakso T. Yetukuri L. Backhed F. Hanninen V. (2009). Gut microbiota affects lens and retinal lipid composition. Exp. EYE Res. 89, 604–607. doi: 10.1016/j.exer.2009.06.018 19591827

[B80] Parthasarathy R. Santiago F. Mccluskey P. Kaakoush N. O. Tedla N. Wake D. (2023). The microbiome in HLA-B27-associated disease: implications for acute anterior uveitis and recommendations for future studies. Trends IN Microbiol. 31, 142–158. doi: 10.1016/j.tim.2022.08.008 36058784

[B81] Pickard J. M. Zeng M. Y. Caruso R. Núñez G. (2017). Gut microbiota: Role in pathogen colonization, immune responses, and inflammatory disease. Immunol. Rev. 279, 70–89. doi: 10.1111/imr.12567 28856738 PMC5657496

[B82] Rinninella E. Mele M. C. Merendino N. Cintoni M. Anselmi G. Caporossi A.. (2018). The role of diet, micronutrients and the gut microbiota in age-related macular degeneration: new perspectives from the gut-retina axis. Nutrients 10 (11), 1677. doi: 10.3390/nu10111677 30400586 PMC6267253

[B83] Robles Alonso V. Guarner F. (2013). Linking the gut microbiota to human health. Br. J. Nutr. 109 Suppl 2, S21–S26. doi: 10.1017/S0007114512005235 23360877

[B84] Rosenbaum J. T. Asquith M. (2018). The microbiome and HLA-B27-associated acute anterior uveitis. Nat. Rev. Rheumatol. 14, 704–713. doi: 10.1038/s41584-018-0097-2 30301938 PMC6597169

[B85] Rosenbaum J. T. Kim H. W. (2013). Innate immune signals in autoimmune and autoinflammatory uveitis. Int. Rev. OF Immunol. 32, 68–75. doi: 10.3109/08830185.2012.750132 23360159

[B86] Rosenbaum J. T. Lin P. Asquith M. (2016). The microbiome, HLA, and the pathogenesis of uveitis. JAPANESE J. OF Ophthalmol. 60, 1–6. doi: 10.1007/s10384-015-0416-y 26370944

[B87] Rowan S. Francisco S. G. Tsai C. F. Barger K. Smith D. Zhou C. X.. (2021). Manipulation of gut microbiota affects diet- and age-related retinal degeneration. Invest. Ophthalmol. Visual Sci. 62.

[B88] Rowan S. Jiang S. H. Korem T. Szymanski J. Chang M. L. Szelog J.. (2017). Involvement of a gut-retina axis in protection against dietary glycemia-induced age-related macular degeneration. Proc. Natl. Acad. Sci. U. S. A. 114, E4472–E4481. doi: 10.1073/pnas.1702302114 28507131 PMC5465926

[B89] Scanlan P. D. Shanahan F. O’mahony C. Marchesi J. R. (2006). Culture-independent analyses of temporal variation of the dominant fecal microbiota and targeted bacterial subgroups in Crohn’s disease. J. Clin. Microbiol. 44, 3980–3988. doi: 10.1128/JCM.00312-06 16988018 PMC1698357

[B90] Schaefer L. Hernandez H. Coats R. A. Yu Z. Y. Pflugfelder S. C. Britton R. A.. (2022a). Gut-derived butyrate suppresses ocular surface inflammation. Sci. Rep. 12, 4512. doi: 10.1038/s41598-022-08442-3 35296712 PMC8927112

[B91] Schaefer L. Trujillo-Vargas C. M. Midani F. S. Pflugfelder S. C. Britton R. A. De Paiva C. S. (2022b). Gut microbiota from sjogren syndrome patients causes decreased T regulatory cells in the lymphoid organs and desiccation-induced corneal barrier disruption in mice. Front. IN Med. 9, 852918. doi: 10.3389/fmed.2022.852918 PMC8959809 35355610

[B92] Sekirov I. Russell S. L. Antunes L. C. Finlay B. B. (2010). Gut microbiota in health and disease. Physiol. Rev. 90, 859–904. doi: 10.1152/physrev.00045.2009 20664075

[B93] Sharma S. M. Jackson D. (2017). Uveitis in the spondyloarthopathies. Best Pract. Res. IN Clin. Rheumatol. 31, 846–862. doi: 10.1016/j.berh.2018.08.002 30509444

[B94] Shi N. Li N. Duan X. Niu H. (2017). Interaction between the gut microbiome and mucosal immune system. Mil Med. Res. 4, 14. doi: 10.1186/s40779-017-0122-9 28465831 PMC5408367

[B95] Shivaji S. (2017). We are not alone: a case for the human microbiome in extra intestinal diseases. GUT Pathog. 9, 13. doi: 10.1186/s13099-017-0163-3 28286571 PMC5339978

[B96] Su X. Yin X. Liu Y. Yan X. Zhang S. Wang X.. (2020). Gut dysbiosis contributes to the imbalance of treg and th17 cells in Graves’ Disease patients by propionic acid. J. Clin. Endocrinol. Metab. 105 (11), dgaa511. doi: 10.1210/clinem/dgaa511 32785703

[B97] Szablewski L. (2018). Human gut microbiota in health and Alzheimer’s disease. J. Alzheimers Dis. 62, 549–560. doi: 10.3233/JAD-170908 29480188

[B98] Tang W. H. Kitai T. Hazen S. L. (2017). Gut microbiota in cardiovascular health and disease. Circ. Res. 120, 1183–1196. doi: 10.1161/CIRCRESAHA.117.309715 28360349 PMC5390330

[B99] Thakur P. S. Aggarwal D. Takkar B. Shivaji S. Das T. (2022). Evidence suggesting the role of gut dysbiosis in diabetic retinopathy. Invest. Ophthalmol. Visual Sci. 63, 21. doi: 10.1167/iovs.63.8.21 PMC9339698 35877085

[B100] Thomas A. S. Lin P. (2016). Ocular manifestations of inflammatory bowel disease. Curr. Opin. IN Ophthalmol. 27, 552–560. doi: 10.1097/ICU.0000000000000310 27585211

[B101] Thompson-Chagoyán O. C. Maldonado J. Gil A. (2005). Aetiology of inflammatory bowel disease (IBD): role of intestinal microbiota and gut-associated lymphoid tissue immune response. Clin. Nutr. 24, 339–352. doi: 10.1016/j.clnu.2005.02.009 15896420

[B102] van Eck N. J. Waltman L. (2010). Software survey: VOSviewer, a computer program for bibliometric mapping. Scientometrics 84, 523–538. doi: 10.1007/s11192-009-0146-3 20585380 PMC2883932

[B103] van Nood E. Vrieze A. Nieuwdorp M. Fuentes S. Zoetendal E. G. De Vos W. M.. (2013). Duodenal infusion of donor feces for recurrent Clostridium difficile. N Engl. J. Med. 368, 407–415. doi: 10.1056/NEJMoa1205037 23323867

[B104] Virili C. Stramazzo I. Centanni M. (2021). Gut microbiome and thyroid autoimmunity. Best Pract. Res. Clin. Endocrinol. Metab. 35, 101506. doi: 10.1016/j.beem.2021.101506 33648848

[B105] Vural M. Gilbert B. Ustun I. Caglar S. Finckh A. (2020). Mini-review: human microbiome and rheumatic diseases. Front. Cell. Infection Microbiol. 10. doi: 10.3389/fcimb.2020.491160 PMC7693548 33304855

[B106] Wang X. Yu C. F. Liu X. M. Yang J. S. Feng Y. L. Wu Y. J.. (2022). Fenofibrate ameliorated systemic and retinal inflammation and modulated gut microbiota in high-fat diet-induced mice. Front. Cell. INFECTION Microbiol. 12, 839592. doi: 10.3389/fcimb.2022.839592 PMC9201033 35719341

[B107] Watane A. Cavuoto K. M. Rojas M. Dermer H. Day J. O. Banerjee S.. (2022). Fecal microbial transplant in individuals with immune-mediated dry eye. Am. J. OF Ophthalmol. 233, 90–100. doi: 10.1016/j.ajo.2021.06.022 34214453 PMC8678170

[B108] Yang G. Wei J. L. Liu P. Y. Zhang Q. H. Tian Y. Hou G. W.. (2021). Role of the gut microbiota in type 2 diabetes and related diseases. METABOLISM-CLINICAL AND Exp. 117, 154712. doi: 10.1016/j.metabol.2021.154712 33497712

[B109] Ye Z. Wu C. Y. Zhang N. Du L. P. Cao Q. F. Huang X. Y.. (2020). Altered gut microbiome composition in patients with Vogt-Koyanagi-Harada disease. GUT Microbes 11, 539–555. doi: 10.1080/19490976.2019.1700754 31928124 PMC7524263

[B110] Zamvil S. S. Spencer C. M. Baranzini S. E. Cree B. (2018). The gut microbiome in neuromyelitis optica. Neurotherapeutics 15, 92–101. doi: 10.1007/s13311-017-0594-z 29280091 PMC5794705

[B111] Zarate-Blades C. R. Horai R. Mattapallil M. J. Ajami N. J. Wong M. Petrosino J. F.. (2017). Gut microbiota as a source of a surrogate antigen that triggers autoimmunity in an immune privileged site. GUT Microbes 8, 59–66. doi: 10.1080/19490976.2016.1273996 28045579 PMC5361604

[B112] Zhang J. Y. Xie B. Q. Barba H. Nadeem U. Movahedan A. Deng N. N.. (2022). Absence of gut microbiota is associated with RPE/choroid transcriptomic changes related to age-related macular degeneration pathobiology and decreased choroidal neovascularization. Int. J. Mol. Sci. 23 (17), 9676. doi: 10.3390/ijms23179676 36077073 PMC9456402

[B113] Zhuang Z. Wang Y. Zhu G. Gu Y. Mao L. Hong M.. (2017). Imbalance of Th17/Treg cells in pathogenesis of patients with human leukocyte antigen B27 associated acute anterior uveitis. Sci. Rep. 7, 40414. doi: 10.1038/srep40414 28091550 PMC5238419

[B114] Zinkernagel M. S. Zysset-Burri D. C. Keller I. Berger L. E. Leichtle A. B. Largiader C. R.. (2017). Association of the intestinal microbiome with the development of neovascular age-related macular degeneration. Sci. Rep. 7, 40826. doi: 10.1038/srep40826 28094305 PMC5240106

